# Exosome-related genes influence the progression of stroke through neuroinflammatory responses

**DOI:** 10.3389/fneur.2025.1517588

**Published:** 2025-05-19

**Authors:** Boyan Zhao, Jianing Wu, Mingyang Han, Xuan Rong, Jin Jin, Shiya Liu, Cheng Zhang, Ruotian Zhang, Xin Chen, Fei Peng, Xingli Dong, Shiguang Zhao

**Affiliations:** ^1^Department of Neurosurgery, Shenzhen University General Hospital, Shenzhen, Guangdong, China; ^2^School of Medicine, Shenzhen University, Shenzhen, Guangdong, China; ^3^University of Toronto Scarborough, Toronto, ON, Canada; ^4^Department of Neurosurgery, The First Affiliated Hospital of Harbin Medical University, Harbin, China; ^5^Department of Neurosurgery and Neurosurgical Disease Research Centre, The Second Affiliated Hospital of Guangzhou Medical University, Guangzhou, Guangdong, China; ^6^Central Laboratory, Shenzhen University General Hospital, Shenzhen, Guangdong, China

**Keywords:** ischemic stroke, bioinformatics, neuroinflammation, exosome, immune infiltration, single-cell RNA sequencing

## Abstract

Ischemic stroke (IS) ranks among the top causes of mortality and disability globally. Exosomes exert a crucial effect on maintaining a complex regulatory balance with neuroinflammation in IS. Hence, this research aimed to elucidate the roles of exosome-related genes IS. We integrated data from five IS-related datasets from the Gene Expression Omnibus (GEO) database and exosome-related genes from ExoCarta. The least absolute shrinkage and selection operator regression and random forest models were performed to detect feature genes. Search Tool for the Retrieval of Interacting Genes and Cytoscape were employed to recognize the hub genes. Enrichment analyses were conducted to examine biological processes. CIBERSORT and MCPcounter were applied to assess immune infiltration, and Principal Component Analysis was utilized to explore the associations of feature genes and hub genes with immune cells. After identified different cell types, we analyzed differentiation, developmental trajectory, and interactions of the cell populations. Middle cerebral artery occlusion models were conducted on mice, followed by quantitative polymerase chain reaction to assess the expression levels of each hub gene. We identified 13 feature genes and 10 hub genes. Through qPCR, LGALS3, CD36, TLR2, ICAM1, and CD14 were significantly upregulated after Middle Cerebral Artery Occlusion surgery. Hub genes were significantly involved in inflammatory responses, as well as chemokine signaling and JAK–STAT signaling. Immuno-infiltration analysis revealed significant differences in immune cell populations between IS and controls. Additionally, neutrophils and monocytes/macrophages were positively correlated with CD14 and LGALS3, respectively. Single-cell analysis revealed 19 cell subpopulations with detailed pseudo-time trajectory predictions, highlighting the developmental importance of MG2 microglial cells. In conclusion, our results illuminate exosomal genes, including LGALS3 and CD14, participate in the progression of IS through neuroinflammation, as well as highlight potential therapeutics to mitigate IS injury.

## 1 Introduction

Globally, ischemic stroke (IS) is a primary cause of morbidity and mortality, comprising about 87% of all stroke occurrences (1). Annually, approximately 795,000 individuals in the United States suffer a new or recurrent stroke (2). The high incidence and severe outcomes associated with IS underscore the urgent need for an improved understanding and management of this condition. Despite advancements in medical treatments and preventive strategies, mortality rates remain concerning, with 8–12% mortality within 30 days and nearly 20% within 1 year (3). Current therapeutic interventions, including thrombolysis and mechanical thrombectomy, are time-sensitive and have limited efficacy, particularly in cases where treatment is delayed. Consequently, there is a pressing need to enhance early diagnosis and treatment outcomes.

Exosomes are small extracellular vesicles that mediate intercellular communication and are associated with various pathological processes, including inflammation, coagulation, and neural injury (4). In the context of IS, exosomal miRNAs have shown promise as biomarkers. For instance, specific exosomal miRNAs have been identified as differentially expressed in patients with IS, relating to disease severity and outcomes (5). Furthermore, exosomal proteins associated with the coagulation and inflammation have emerged as potential therapeutic targets (6). These investigations highlight the potential of exosomes as carriers for drug delivery, biomarkers for diagnosis, and modulators of immune responses. For example, a strategy has been proposed to design and prepare exosomes from anti-CHAC1 adipose-derived mesenchymal stem cells to inhibit ferroptosis and reduce ischemia/reperfusion (I/R) injury (7). Similarly, curcumin-containing exosomes are capable of reducing damage in lesions, lowering the expression of inflammatory and excitatory amino acid receptors, and promoting neurovascular recovery (8). However, considering the complex crosstalk between exosomes and inflammatory responses, the mechanisms underlying their interactions remain unclear. Currently, significant breakthroughs in the use of exosomes for targeted therapy in IS are lacking, indicating that extensive research in this area is still required.

Following IS, secondary neuroinflammation emerges (9), which can exacerbate damage and contribute to cell death. Conversely, it may also facilitate recovery processes. Current studies have revealed that neurons, glial cells, and vascular components collectively constitute a functional “neurovascular unit” (10). In the aftermath of an IS event, microglia and astrocytes are activated within hours, leading to the generation of cytokines and chemokines, which subsequently promote leukocyte infiltration (11). More specifically, activated microglia secrete inflammatory factors, including cytokines, and enhance their phagocytic functions. This activation contributes positively by enhancing the production of growth factors and facilitating the clearance of necrotic tissue and ischemic debris (12). Nevertheless, the emission of other inflammatory cytokines, including nitric oxide and ROS, can adversely affect brain tissue following ischemia (13). Given this paradoxical mechanism, further research is required to clarify the intricate balance of this dual role, which will enhance our understanding and facilitate the development of effective targeted therapies.

In this research, we sought to uncover the molecular mechanisms contributing to IS by integrating multiomics data with advanced analytical methods. We concentrated on the involvement of exosome-related genes in IS and highlighted the importance of neuroinflammation.

## 2 Materials and methods

**Figure 1:**
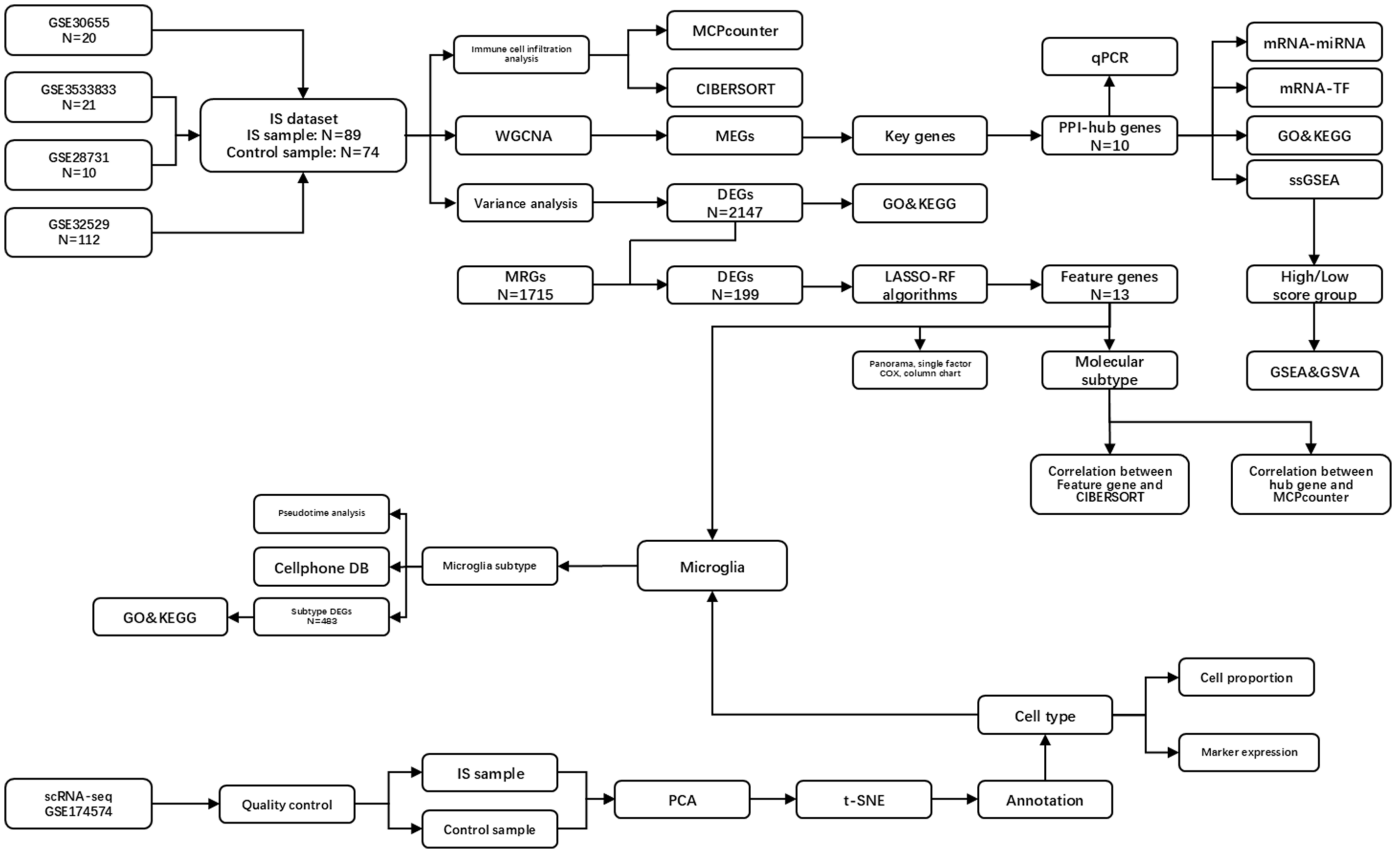
Overall analysis flow chart. IS, ischemic stroke; PPI, protein–protein interaction network; DEGs, differentially expressed genes; ERGs, exosome-related genes; GO, gene ontology; MCPcounter, microenvironment cell population-counter; WGCNA, weighted gene co-expression network analysis; MEGs, module genes; KEGG, Kyoto encyclopedia of genes and genomes; LASSO, least absolute shrinkage and selection operator; RF, random forest; PCA, principal component analysis; t-SNE, t-distributed stochastic neighbor embedding; GSEA, gene set enrichment analysis; GSVA, gene set variation analysis.

Overall analysis flow chart is shown in Figure 1.

### 2.1 IS dataset processing and difference analysis

Using the GEOquery 2.70 package (14), we retrieved the IS datasets GSE30655, GSE35338 (15), GSE28731, and GSE32529 (16) from the GEO database (17).^1^ These datasets, sourced from *Mus musculus*, were all based on the GPL1261. The GSE30655 dataset comprises 14 ischemic samples and 6 control samples. The GSE35338 comprises 11 stroke samples and 10 control samples. The GSE28731 dataset comprises six ischemic samples and four control samples. The GSE32529 dataset comprises 58 ischemic samples and 54 control samples. In total, 74 control samples and 89 ischemic samples were included. The batch effect was addressed using the sva package (18). Subsequently, the limma package (19) was implemented to normalize the combined dataset, leading to the creation of the IS dataset.

We downloaded the ischemic stroke-related single-cell dataset GSE174574 (20) from the GEO database. The data source is *Mus musculus*, and its data platform is GPL21103. Three control samples (GSM5319987, GSM5319988, and GSM5319989) and three ischemic samples (GSM5319990, GSM5319991, and GSM53199892) were used for subsequent analyses.

In terms of single-cell data, we employed the R package Seurat 4.0 (21) to construct single-cell data as Seurat objects, as well as the PercentageFeatureSet in the Seurat package to estimate the percentage of mitochondria in each cell. When the ratio of mitochondrial genes in a cell is excessively high, the cell may be undergoing apoptosis or lysis. Therefore, we filtered out cells with >5% mitochondrial gene content. Furthermore, since low-quality cells or empty droplets typically contain few genes, and doublets often exhibit an abnormally high number of genes, we excluded cells with fewer than 200 features. Furthermore, cells contained over than 20,000 intracellular UMI were excluded. The data obtained after the above quality control procedures were used for later investigations.

Finishing the quality control procedure for the Seurat subjects, we performed linear dimension reduction and calculated the Principal Component utilizing the most variable expression of the genes (22). Then, we used Seurat’s “FindNeighbors” and “FindClusters” functions to group the cells into the optimal number of clusters for cell type identification. Subsequently, t-SNE (23) was performed to project the information from the selected principal components into two dimensions, facilitating graph-based visual clustering of the cells. Mouse exosome-related genes were acquired from Exo-Carta^2^ (24) for subsequent analyses, which includes 1715 mouse exosome-related genes.

### 2.2 Differential analysis

We applied the limma package (version 3.58.1) for differential analysis between ischemic and controls in the IS dataset in order to obtain differentially expressed genes (DEGs). DEGs were selected for further analysis if they met the criteria of an absolute log fold change (|logFC|) greater than 0.25 and a *p*-value of less than 0.05, and visualizations of the results were displayed as a volcano plot and a heatmap presented with the R package ggplot2 generated and the R package pheatmap version 1.0.12, respectively.

### 2.3 Gene ontology and Kyoto encyclopedia of genes and genomes enrichment analysis

GO (25) analysis is frequently employed for large-scale functional enrichment studies. KEGG (26) is a widely utilized database. GO and KEGG enrichment analyses were conducted by the clusterProfiler package (version 4.2.0) in R (27). A false discovery rate cutoff value of <0.05 was regarded as statistically significant. The top eight results with the lowest *p*-values in GO, as well as the top eight results with the lowest *p*-values in KEGG are shown in the bar graph.

### 2.4 Acquisition of exosome-related genes

Identifying exosomal genes within the DEGs in mice, we obtained 1715 mouse exosome-related genes from ExoCarta. We displayed the overlap between exosomal genes and DEGs in a Venn diagram. Totally, 199 differentially expressed exosome-related genes in the mouse IS dataset were extracted for subsequent analyses.

### 2.5 Feature genes based on the least absolute shrinkage and selection operator regression model

The LASSO regression model is distinguished by variable selection and complexity adjustment while fitting a generalized linear model. Regularization involves applying a shrinkage penalty to constrain the coefficients. This process utilizes the sum of the absolute values of all feature weights, enhancing the interpretability of the model. The LASSO regression model was performed by the glmnet 4.1.8 package (28) for genes with *p* < 0.05 in univariate COX analysis. The model construction process included screening features for inclusion, selecting only those that contributed most significantly.

### 2.6 Feature genes were screened based on a random forest model

RF (29) serves as an ensemble learning algorithm according to decision trees. In random forest, multiple decision trees are constructed, and the final prediction result is obtained by voting or taking the average of each decision tree. In a random forest, each decision tree is trained on multiple “bootstrap” samples from the original dataset created by sampling with replacement. At the same time, at each node, the random forest randomly selects a subset of features for splitting, which helps reduce feature correlation and improves the model’s generalization ability. In constructing the random forest model, each gene was regarded as a feature. We calculated the frequency of each gene used as a split node in the construction of multiple decision trees to determine its contribution to classification results. Genes with high importance scores are likely to be related to the disease. The selected feature genes were then intersected with those identified by the LASSO model, and univariate COX analysis was executed for these feature genes to generate a comprehensive gene profile.

### 2.7 Identification of key genes

Weighted Gene Co-expression Network Analysis (WGCNA) aims to test co-expressed gene modules (30), investigate the connections between gene networks and phenotypes, and analyze the core genes in the network. Therefore, we first calculated the soft threshold using the pickSoftTreshold function for exosome-related genes, with three being the best soft threshold. We subsequently constructed a scale-free network according to a soft threshold, constructed a topological matrix, as well as conducted hierarchical clustering. With a minimum requirement of 100 genes per module, we dynamically cut and identified the gene modules to calculate Eigengenes. The modules with a correlation above 0.5 were combined, leading to the identification of four final modules. We performed Pearson correlation analysis to test the relation between modules and IS, leading to the identification of MEGs. These were subsequently screened as the key genes for this study.

### 2.8 Identifications of PPI network analysis and hub gene

The intersection of DEGs and MEGs was used for PPI analysis. We used the STRING^3^ (31) to establish PPI networks for selected genes. Key interacting genes were extracted from the STRING database to formulate a network model, which was visualized by Cytoscape (32). Additionally, the plugin CytoHubba (33) from Cytoscape was employed to analyze the hub genes within network.

### 2.9 Construct mRNA-miRNA and mRNA-TF interaction networks

The Encyclopedia of RNA Interactomes (ENCORI) database^4^ (34), is a resource for exploring interactions involving microRNAs, non-coding RNAs (ncRNAs), and RNA-binding proteins (RBPs). This database provides data on interactions between microRNAs and ncRNAs, microRNAs and mRNAs, ncRNAs and RNAs, and RBPs with ncRNAs and mRNAs. These interactions are from CLIP-seq and degradome sequencing data, which include plant-specific analyses, and the database offers visual tools for investigating microRNA targets. Through miRDB database, target genes of miRNAs and functional annotation (35) were established. Additionally, we employed the ENCORI and miRDB databases to pinpoint miRNAs that engage with hub genes. We overlapped the mRNA-miRNA data to create the mRNA-miRNA interaction network.

The CHIPBase (36) database (version 2.0)^5^ utilizes DNA-binding protein ChIP-seq data to identify binding site matrices and their corresponding sequences. Additionally, it elucidates the relationships in transcriptional regulation between TFs and corresponding genes. We searched both the CHIPBase (version 3.0) and hTF target databases for TFs binding to hub genes and presented them by the Cytoscape software.

### 2.10 Calculate the ischemic stroke and exosome-related gene score based on the IS dataset

The relative abundance of individual gene within the dataset sample was quantified using the single-sample gene set enrichment analysis (ssGSEA). The R package GSVA (37) was performed to estimate the hub gene scores for ischemic samples in the IS dataset derived from the hub gene expression matrix utilizing the ssGSEA algorithm. Subsequently, samples were categorized into high- and low-scoring groups according to the median score, which were then analyzed using GSEA and GSVA.

### 2.11 Gene set enrichment analysis and gene set variation analysis

We utilized GSEA (38) to identify variations in biological process (BP) among different groups. GSEA serves as a computational technique to analyze the statistical differences of a specific group of genes between two distinct biological conditions. It is widely for estimating the alterations in pathways and BP activity in samples from expression datasets. GSEA analysis was executed by the R package clusterProfiler, and a *p*-value<0.05 was defined as significantly enriched.

GSVA is used for gene set enrichment analysis of nonduplicate samples. Using GSVA, the score of the relevant gene set might be calculated, and the differential pathway scores of each sample could be analyzed to identify the differentially expressed pathways among the groups. We acquired the reference gene set “mh. All. V2023.1. Mm. Symbols” from the MSigDB database and utilized the GSVA to analyze common pathways. We employed the limma package to estimate score disparities between the two groups for various pathways, setting the parameter to *p*-value <0.05. Finally, a heatmap was generated to represent these differences.

### 2.12 Immune infiltration analysis based on CIBERSORT

CIBERSORT^6^ is an R tool (39) that utilizes linear support vector regression to disentangle the expression matrix of human or murine immune cell subtypes. This method relies on a recognized reference collection that offers a gene expression signature for 24 immune cell subtypes, enabling the calculation of immune cell infiltration. Interactions among immune cells can affect the immune pathways and functions within the BP of the immune system. Hence, we obtained immune infiltration based on the IS dataset and utilized the ggplot2 to construct a bar chart illustrating the dispersion of immune cell infiltration. Additionally, we generated a correlation heat map to reflect the correlation among immune cells and between prognostic genes and immune cells. Finally, we contrasted the scores of different immune cells between patients with IS and controls to pinpoint immune cells exhibiting varying levels of infiltration between the two cohorts.

### 2.13 Immune infiltration analysis based on MCPcounter

MCPcounter (40) was used to quantify the absolute abundances of eight immune cells and two stromal cells using the IS dataset expression matrix. For samples in each IS dataset, the abundance score was computed as the geometric mean of the gene expression values specific to each cell type, which were calculated independently for each sample.

### 2.14 Construct molecular subtypes based on disease-feature genes

Consensus clustering (41) was used to ascertain the quantity and membership of potential clusters. ConsensusClusterPlus (42) was employed for consensus clustering on the IS dataset, aiding the identification of distinct IS disease subtypes. Additionally, we performed Principal Component Analysis to analyze the different molecular subtypes of IS and to evaluate the associations among hub genes, feature genes, and immune cells across these subtypes.

### 2.15 Cell annotation

For Seurat objects with single-cell data, 19 clusters were visualized using t-sne. Artificial annotation of cell type marker genes identified 12 different cell types, containing vascular smooth muscle cells (SMC); perivascular fibroblast-like cells (FB); CNS border-associated macrophages (CAM); monocyte-derived cells (MdCs); endothelial cells (EC); ependymocytes (EPC); microglia (MG); neutrophils (NEUT); astrocytes (ASC); oligodendrocytes (OLG); lymphocytes (LYM); and pericytes (PC).

For the 12 annotated cell clusters, we calculated the DEGs between all cell clusters using the function “FindAllMarkers.” We selected genes based on the benchmarks of |log2FoldChange| > 0.1 and *p*-value <0.05, which we designated as our single-cell DEGs for further analysis.

### 2.16 AddModuleScore scored the cell population

The AddModuleScore (43) was employed to compute the scores for cell types or BP according to gene expression data within a single sample. This approach allows us to quantitatively assess the relative abundance of different cell types or the activity levels of certain BP. The core concept of AddModuleScore involves defining a set of genes associated with each cell type or BP in advance and aggregating the expression values of these genes to obtain a single score that reflects the activity of that cell type or BP. We used hub genes as reference genes to calculate scores for different cell populations and ascertained the cell subset with the highest score as microglial cells for subsequent analysis.

### 2.17 Pseudo-time series analysis

A pseudo-time (44) analysis was employed to arrange cells along a trajectory according to the temporal sequence of their gene expression profiles. This method segments the sample into various differentiation states according to the gene expression patterns, generating an intuitive lineage development dendrograph that can predict cell differentiation and development trajectories. The results of pseudo-time analysis confirm the origin and endpoint of differentiation according to the trajectory allocation of cell types as well as expression changes of feature genes. For a subset of epithelial cells, we performed pseudo-time analysis to predict the developmental trajectories of the subpopulation of cells and analyze the changes in RNA modification-related genes over time.

### 2.18 Cellular communication analysis

Cytokines and membrane proteins facilitate communication among multicellular organisms, exerting a critical effect on modulating vital biological processes and ensuring the organism operates efficiently and in an orderly manner. Receptor-ligand-mediated intercellular communication is essential for the coordination of diverse BP, such as development, differentiation, and disease. Cell communication analysis determines interactions between different cells by counting the expression and pairing of receptors and ligands in different cell types. The R package CellPhoneDB (45) was used for cell communication analysis. CellPhoneDB mainly obtains interactions among different cell types by analyzing the expression profiles of receptors in one type of cell and ligands in another. CellPhoneDB not only contains the database-annotated receptors and ligands but also provides artificially annotated protein families involved in cell communication, providing the subunit structure of receptors and ligands.

### 2.19 Animals

Wildtype male C57BL/6 mice (4–6 weeks old) were employed in *in vivo* studies. Mice were provided by the Guangdong Provincial Medical Experimental Animal Center. The animal research was conducted in compliance with the National Institutes of Health Guide for the Care and Use of Laboratory Animals, and received approval from the Institutional Animal Care and Use Committee of Shenzhen University Medical School (Approval No.: IACUC-202300110). Mice were maintained under a 12 h light/dark cycle and were provided unrestricted access to both food and water. Isoflurane anesthesia (RWD, Shenzhen, China) was used for performing invasive procedures.

### 2.20 Middle cerebral artery occlusion *in vivo*

The procedure for MCAO was executed according to formerly described methods (46). Concisely, isoflurane (4% induction; 2% maintenance) was used to anesthetize the mice (4% induction, 2% maintenance) throughout the surgery, and the right carotid arteries were surgically exposed. A nylon monofilament, calibrated according to body weight, was inserted via the external carotid artery and progressed into the internal carotid artery, successfully blocking the origin of the middle cerebral artery for a duration of 90 min.

### 2.21 MRI in MCAO mice

An MRI scan was performed while mice were under anesthesia with 1% isoflurane. All images were acquired by a 9.4 T/160 mm animal MRI system (United Imaging 9.4 T MRI), employing a 72 mm quadrature volume coil for excitation and two-channel coils for detection.

### 2.22 Cerebral blood flow measurement

Laser speckle flow imaging was employed to assess CBF after MCAO surgery. Concisely, after successfully anesthetized, mice were sanitized with iodophor before exposing the skull. The overlying fascia was carefully removed to the greatest extent possible, as well as 0.9% saline was applied to preserve the liquid level stable. This technique (RFLSI III, RWD, China) was employed to capture images and quantify CBF in the penumbra region.

### 2.23 Total RNA extraction and fluorescent quantitative PCR

Blood samples were obtained through cardiac puncture, without thoracotomy. Total RNA was obtained from the peripheral blood of the mice utilizing Trizol reagent. A commercial kit was employed for the synthesis of complementary DNA (cDNA), which was then diluted to a consistent concentration and deposited at −20°C. For the quantitative reverse transcription PCR (qRT-PCR), the reaction mixture contained 2 μL of cDNA, 10 μL of SYBR Premix Ex Taq II (Tli RNase H Plus), 1 μL each of forward and reverse primers, and 6 μL of double-distilled water (ddH₂O), resulting in a total volume of 20 μL. The PCR protocol commenced with a preliminary activation phase of the polymerase at a temperature of 95°C for a duration of 30 s. This was succeeded by 40 cycles consisting of a denaturation step at 95°C lasting for 5 s, followed by an annealing and extension phase at 60°C for 34 s. The threshold cycle (Ct) values for both the target gene and GADPH were evaluated utilizing Mx-Pro software (Mx3005p, Agilent, Santa Clara, CA, United States). Relative expression levels were normalized by employing the double ΔCt method for calculation. Primer sequences are listed in the Table 1.

**Table 1:** Primer sequences.

Primer names	Primer sequences (5′-3′)
M-Icam1-S	GTACTGTACCACTCTCAAAATAACTGG
M-Icam1-A	TGGGGCTTGTCCCTTGAGT
M-Tlr2(1)-S	CCAAAGTCTAAAGTCGATCCGC
M-Tlr2(1)-A	AGCCCATTGAGGGTACAGTCGT
M-Tgfb1(2)-S	CCCTGGATACCAACTATTGCTTC
M-Tgfb1(2)-A	AGTAGACGATGGGCAGTGGCT
M-Cd14-S	TCAAGTTCCCGACCCTCCAA
M-Cd14-A	GCCCAGTGAAAGACAGATTGAG
M-Cd36(2)S	GGAACTGTGGGCTCATTGCT
M-Cd36(2)A	CAACTTCCCTTTTGATTGTCTTCTC
M-Cd86(3)-S	AACGTATTGGAAGGAGATTACAGCT
M-Cd86(3)-A	CCTGCTAGGCTGATTCGGCT
M-CD68-S	GCCCAAGGAACAGAGGAAGACT
M-CD68-A	GTGGTGGCAGGGTTATGAGTG
M-Lgals3-S	CCAACGCAAACAGGATTGTTCTA
M-Lgals3-A	TGATTTCCCGGAGGTTCTTCAT
M-Csf1(1)-S	CAGGAGTATTGCCAAGGAGGTG
M-Csf1(1)-A	AGCGCATGGTCTCATCTATTATGTC
M-FN1(RZ)-S	AAGGCTGGATGATGGTGGACT
M-FN1(RZ)-A	TCGGTTGTCCTTCTTGCTCC
M-GAPDH-S	CCTCGTCCCGTAGACAAAATG
M-GAPDH-A	TGAGGTCAATGAAGGGGTCGT

### 2.24 Statistical analysis

Data processing and statistical evaluations were performed utilizing R programming software (version 4.1.2, available at https://www.r-project.org/). To compare continuous variables across the two groups, an independent Student’s *t*-test was employed for those variables exhibiting a normal distribution. Conversely, for variables that did not conform to a normal distribution, the Mann–Whitney U test, also known as the Wilcoxon rank-sum test, was utilized. All statistical *p*-values were calculated as two-sided. A *p*-value threshold of less than 0.05 was established to determine statistical significance.

## 3 Results

### 3.1 IS dataset processing and difference analysis

**Figure 2:**
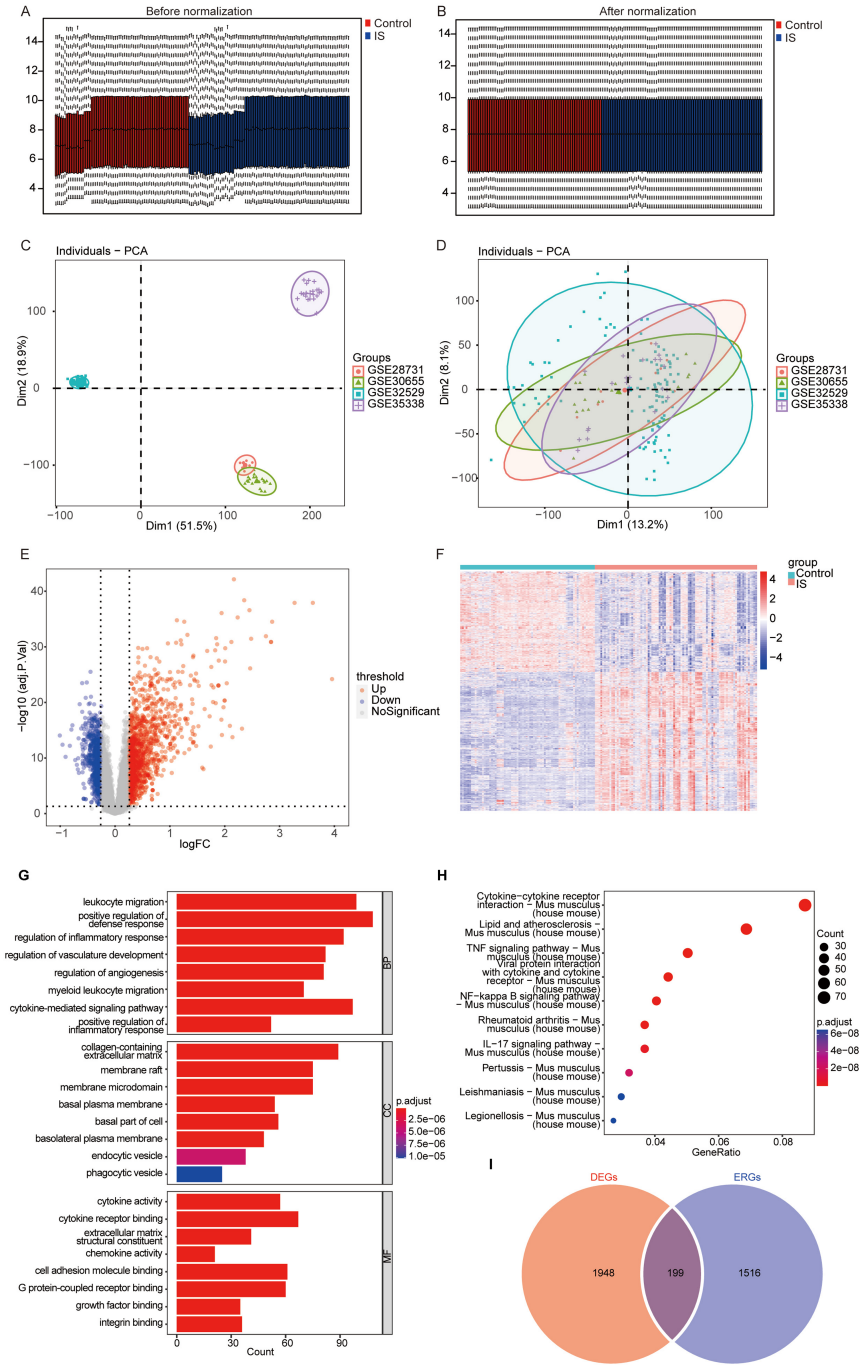
Data preprocessing and difference analysis for GSE30655, GSE353383, GSE28731, and GSE32529. **(A)** Boxplot prior to normalization; **(B)** boxplot subsequent to normalization; **(C)** PCA cluster prior to the elimination of the batch effect.; **(D)** PCA cluster subsequent to the elimination of the batch effect; **(E)** volcano plot of the differential analysis; **(F)** heatmap of DEGs between the ischemic and control groups (DEGs, differentially expressed genes); **(G)** GO enrichment analysis of DEGs; **(H)** KEGG enrichment analysis of DEGs; **(I)** overlap of exosome-related genes and DEGs (DEGs, differentially expressed genes).

Initially, we integrated four datasets associated with IS, specifically GSE30655, GSE353383, GSE28731, and GSE32529. Subsequently, we employed the R package “sva” to eliminate any batch effects present within the data. Following this, we applied the “limma” package to normalize the merged dataset, ultimately generating the IS dataset necessary for further analysis. The IS dataset included 74 control samples and 89 IS samples. A boxplot is used to show the distribution of the samples before and after standardization. Figure 2A shows the samples before standardization, and Figure 2B shows the samples after standardization. Figure 2C shows the Principal Component Analysis (PCA) cluster map before the elimination of the batch effect, and Figure 2D shows the PCA cluster map subsequent to the removal of this effect. The PCA clustering plots indicate a successful mitigation of the batch effect across the data sets.

We conducted differential expression analysis with the limma package to compare IS and control samples. By applying thresholds of *p* < 0.05 and logFC>0.25, we identified 2,147 DEGs between the two groups, shown in Supplementary Table S1. Among them, 1,247 genes demonstrated upregulation, while 900 genes showed downregulation (Figure 2E). The patterns of DEGs between IS and controls were displayed using a heat map, which highlighted significant distinctions in the gene expression patterns between the two groups (Figure 2F).

Finally, GO and KEGG were conducted for 2,147 DEGs. The eight results exhibiting the lowest *p*-values were selected for display. GO analysis revealed that the DEGs were predominantly associated with BP including leukocyte migration, regulation of inflammatory responses, among others. The DEGs were primarily related to cellular component (CC) such as membrane rafts, and membrane microdomains. In terms of molecular function (MF), they were associated with cytokine activity, cytokine receptor binding, and extracellular matrix structural constituents, among others (Figure 2G). The data of complete GO analysis were displayed in Supplementary Table S2. KEGG analysis indicated that the DEGs were mainly associated with several pathways, including lipid and atherosclerosis (*Mus musculus*), and the TNF signaling pathway (*Mus musculus*), among others (Figure 2H). The KEGG enrichment analysis results are presented in Supplementary Table S3.

To investigate the exosome-related genes among the DEGs in mice, we obtained exosome-related genes from Exocarta^7^, which includes a total of 1,715 mouse exosome-related genes. The Overlap of exosome-related genes and DEGs is shown in the Venn diagram (Figure 2I), and, as a result, 199 genes in the mouse IS dataset were obtained for subsequent analyses.

### 3.2 Feature genes were screened using the LASSO regression and RF model based on the IS dataset

**Figure 3:**
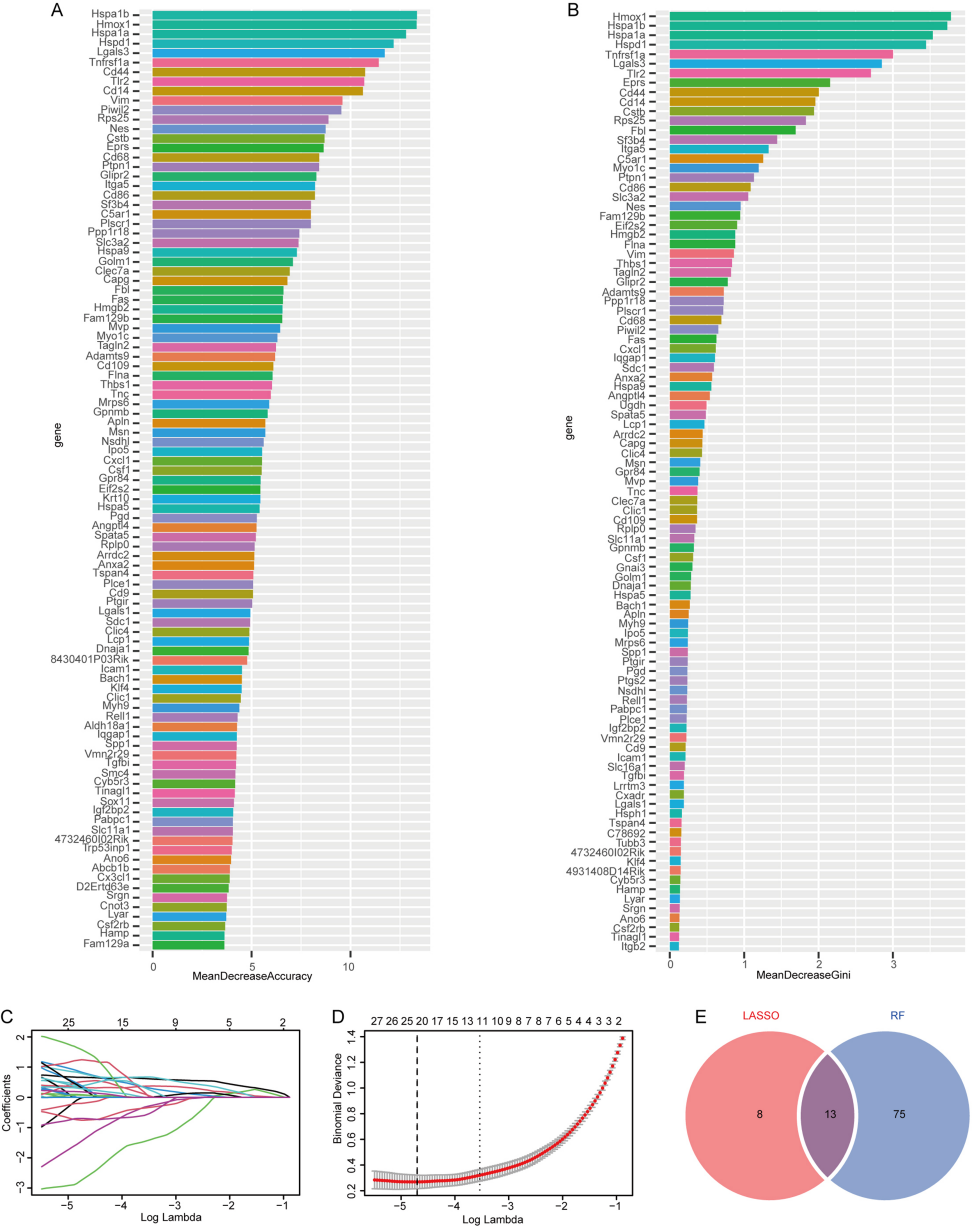
Screening of feature genes using LASSO regression and random forest models based on the IP dataset. **(A)** Average accuracy of feature gene prediction by the RF model; **(B)** the effect of gene heterogeneity predicted by the RF model; **(C)** LASSO regression 10-fold cross-validation plot; **(D)** LASSO regression lambda plot; **(E)** Intersection of the LASSO regression and RF models.

To evaluate the association between exosome-related genes from cerebral ischemia samples and the disease, we used the LASSO regression and RF models to screen the 199 genes identified from the previous analysis. First, the RF model was constructed for dimensionality reduction to predict the mean decrease in accuracy and mean decrease Gini (Figures 3A,B) of the genes, and the genes whose rank was greater than the median importance were selected. Next, we used the LASSO to the IS dataset to simulate and select the number of features, conducted a cross-test on gene coefficients (Figure 3C), determined the optimal Lambda value (Figure 3D), and detected 21 genes in the model as characteristic of IS. Finally, we identified 13 common feature genes as exhibited in a Venn diagram (Figure 3E): HSPA1B, HSPA1A, CD14, LGALS3, PTPN1, FAS, IPO5, CLEC7A, HSPA5, PIWIL2, APLN, NSDHL, and 4732460I02RIK. These genes were used for subsequent analysis.

### 3.3 Univariate COX analysis was conducted to determine whether the feature genes had a significant effect on ischemic stroke

**Figure 4:**
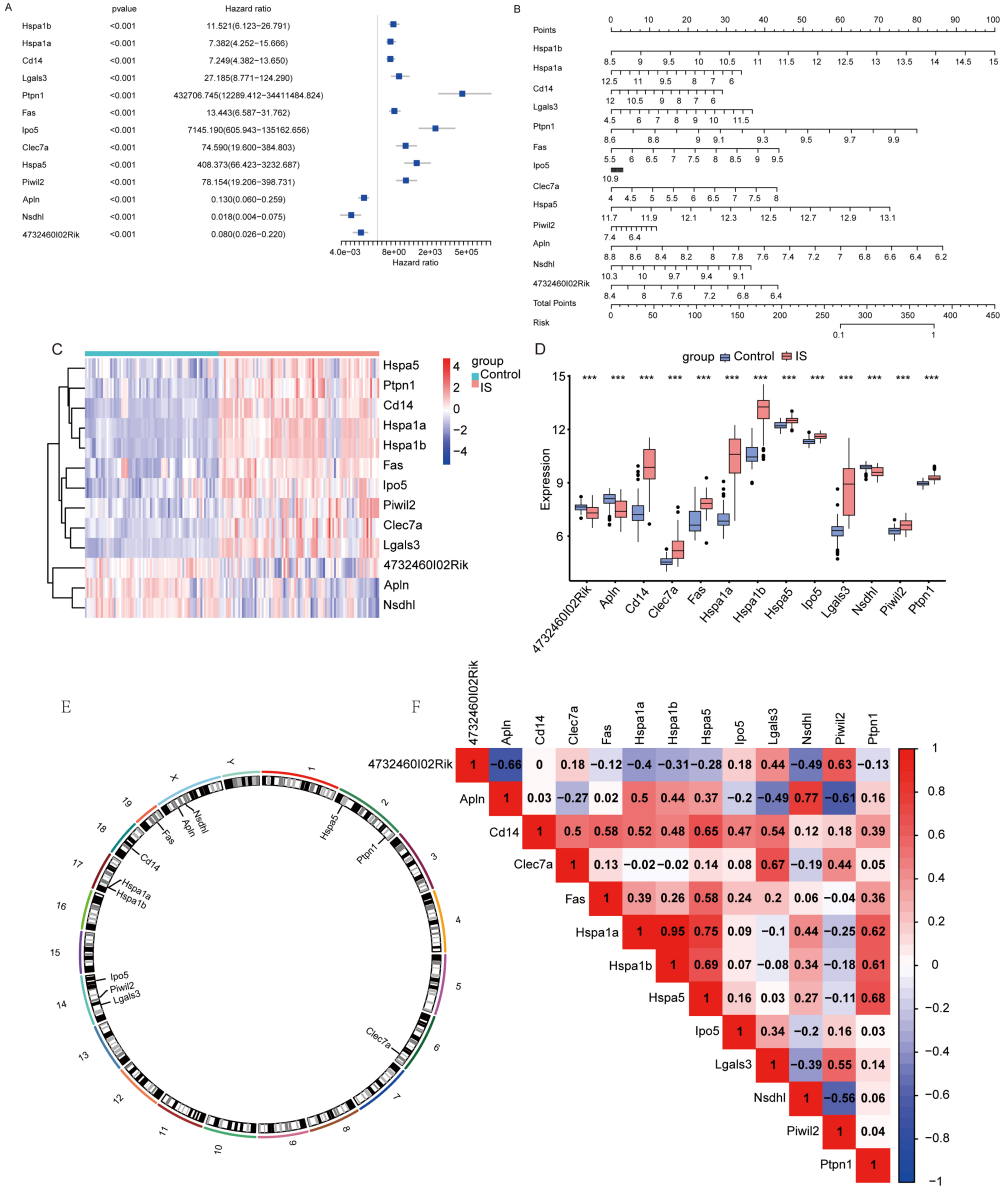
Nomogram for feature genes. **(A)** Univariate COX regression forest plot based on key genes; **(B)** nomogram based on key genes; **(C)** heatmap of feature genes between IS and Control groups; **(D)** difference of feature genes between IS and Control groups (*: *p* < 0.05; * *: *p* < 0.01; ***: *p* < 0.001); **(E)** chromosomal mapping of feature genes; **(F)** heatmap of correlation between feature genes (an absolute correlation coefficient r above 0.8 indicates a strong correlation, while values between 0.3 and 0.8 suggest a weak correlation. Coefficients below 0.3 imply no correlation).

We conducted univariate COX regression on these 13 feature genes in the IS dataset. The analysis revealed that the 13 genes significantly influenced IS outcomes (Figure 4A). Using the rms package, we then created a nomogram according to these 13 feature genes (Figure 4B). A panoramic analysis of the feature genes indicated that HSPA1B, HSPA1A, CD14, LGALS3, PTPN1, FAS, IPO5, CLEC7A, HSPA5, and PIWIL2 were upregulated, while APLN, NSDHL, and 4732460I02RIK were downregulated in the IS group (Figure 4C). The discrepancies in the expression of feature genes are presented in Figure 4D. And Figure 4E shows the localization of the feature genes on the mouse chromosome. HSPA1B and HSPA1A are located on chromosome 17; CD14 on chromosome 18; LGALS3, PIWIL2, and IPO5 on chromosome 14; PTPN1 and HSPA5 on chromosome 2; and FAS on chromosome 19. CLEC7A is located on chromosome 6, APLN and NSDHL are located on chromosome X. Finally, we tested the relationship between the featured genes and visualized them (Figure 4F). The results showed that HSPA1A and HSPA1B had the highest positive association (*r* = 0.95), while the strongest negative association was identified between APLN and 4732460102RIK (*r* = −0.66).

### 3.4 WGCNA of exosome genes

**Figure 5:**
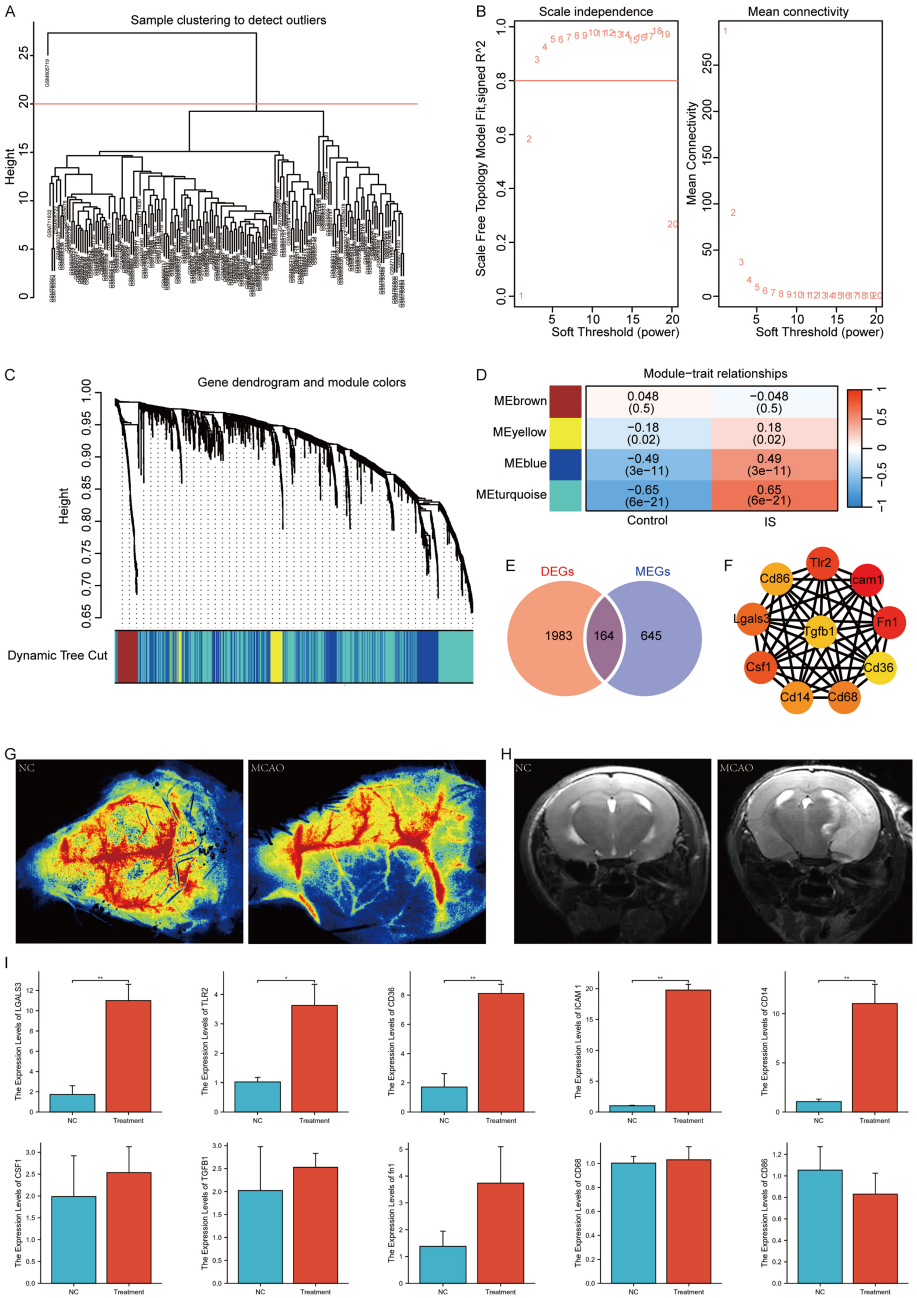
WGCNA analysis according to the IS dataset. **(A)** Sample clustering and outlier removal; **(B)** determination of optimal soft power soft threshold; **(C)** analysis of the aggregation process of module genes; **(D)** heatmap between modules and clinical phenotypes; **(E)** Venn diagram of DEGs and MEGs; **(F)** PPI network of hub genes (where darker colors indicate the importance of nodes); **(G)** CBF assessment of NC and IS mice; **(H)** MRI of NC and IS mice; **(I)** qPCR of hub genes in mice (*: *p* < 0.05; **: *p* < 0.01; ***: *p* < 0.001). NC, negative control.

We performed WGCNA on the IS dataset to identify co-expression modules. First, the samples were clustered (Figure 5A), and the outlier GSM805719 was excluded. A scatter plot analysis indicated that a soft threshold of 3 was optimal for further analysis (Figure 5B). The genes in the IS sample were clustered into four modules: MEbrown, MEyellow, MEblue, and MEturquoise (Figure 5C). Finally, we examined the relationship between the modules and the phenotype of each IS sample using Pearson analysis (Figure 5D) and selected the MEturquoise Module, which exhibited the strongest correlation, for subsequent analysis. The genes within this module, defined as module genes (MEGs), totaled 809. The list is presented in Supplementary Table S4. Subsequently, we formulated a Venn diagram to analyze the overlap between DEGs and MEGs (Figure 5E) and identified 164 key genes, which is detailed shown in Supplementary Table S5. STRING was used to depict the Protein–protein Interaction (PPI) network, which was then imported into the Cytoscape software. The maximal clique centrality algorithm, implemented through the CytoHubba plugin, was utilized to select the top ten genes. The identified hub genes included ICAM1, FN1, CD36, TLR2, CD86, LGALS3, CSF1, CD14, CD68, and TGFB1 (Figure 5F). Subsequently, we performed MCAO surgery on the mice to simulate IS. Following this procedure, CBF (Figure 5G) and MRI (Figure 5H) assessments were performed to confirm the MCAO model. Peripheral blood samples were gathered for the quantitative PCR (qPCR) of hub gene expression. The results indicated significant upregulation of hub genes, including LGALS3, CD36, TLR2, ICAM1, and CD14, in the peripheral blood of MACO mice (Figure 5I).

### 3.5 Regulatory network of hub genes

**Figure 6:**
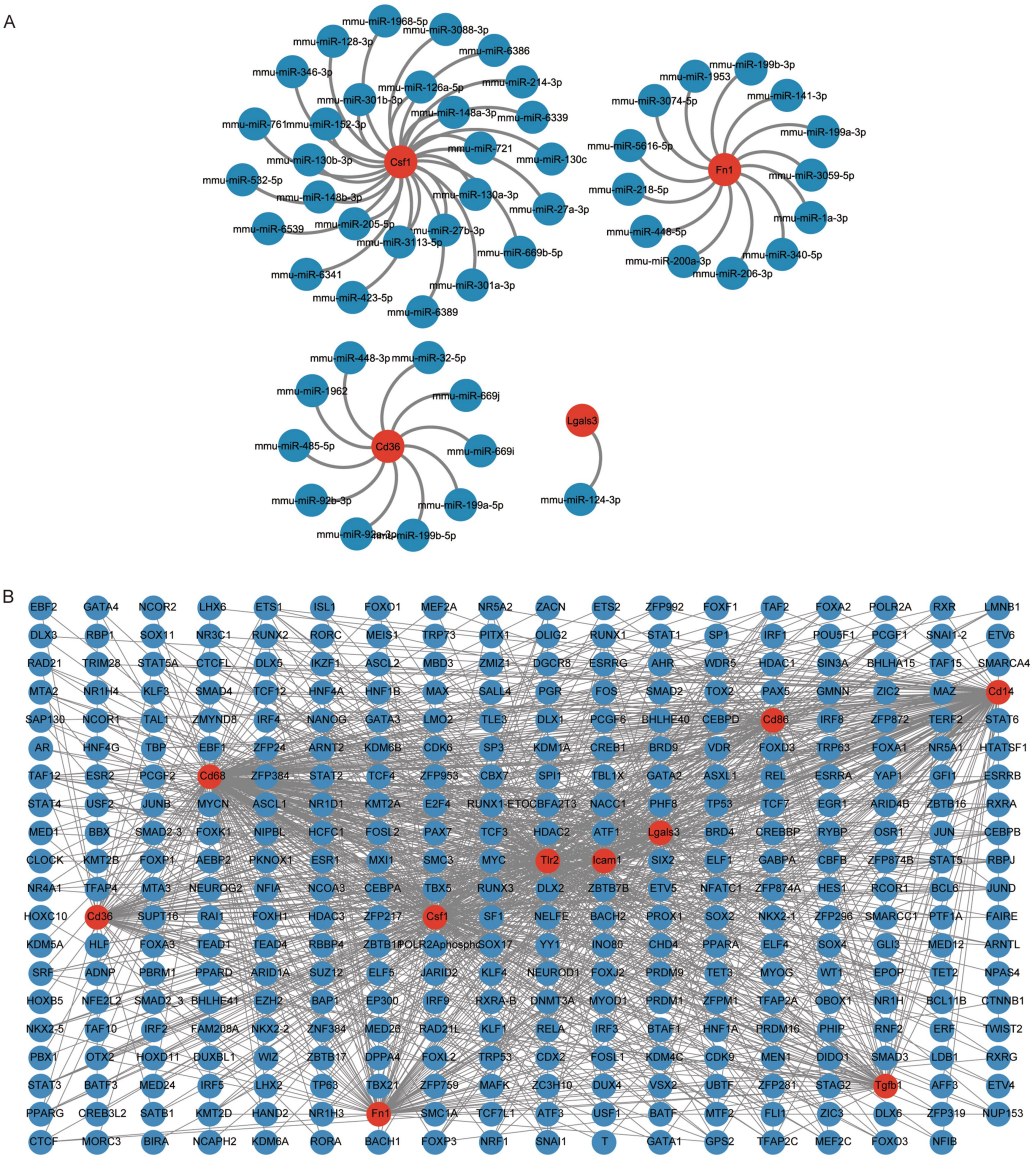
Construction of hub gene regulatory network. **(A)** mRNA-miRNA network of hub genes, where red circular shapes indicate mRNAs and blue circular shapes indicate miRNAs. **(B)** mRNA-TF network of hub genes, where red circular shapes indicate mRNAs and blue circular shapes indicate TFs. TF, transcription factor.

The mRNA-miRNA data was utilized to estimate the miRNAs that related to the 10 hub genes and subsequently the result was visualized through the application of Cytoscape. The mRNA-miRNA interaction network comprised four hub genes (CSF1, FN1, CD36, and LGALS3) and 52 miRNA molecules (Figure 6A). The specific interactions are listed in Supplementary Table S6.

We queried the CHIPBase database (version 3.0) for transcription factors (TFs) binding to the 10 hub genes. Finally, a TF-mRNA interaction network consisting of 10 hub genes (CD14, CD36, CD86, CD68, LGALS3, CSF1, FN1, ICAM1, TLR2, and TGFB1) and 349 TFs was obtained (Figure 6B). The specific relationships are listed in Supplementary Table S7.

### 3.6 Calculate the ischemic stroke & exosome-related gene score based on the IS dataset

**Figure 7:**
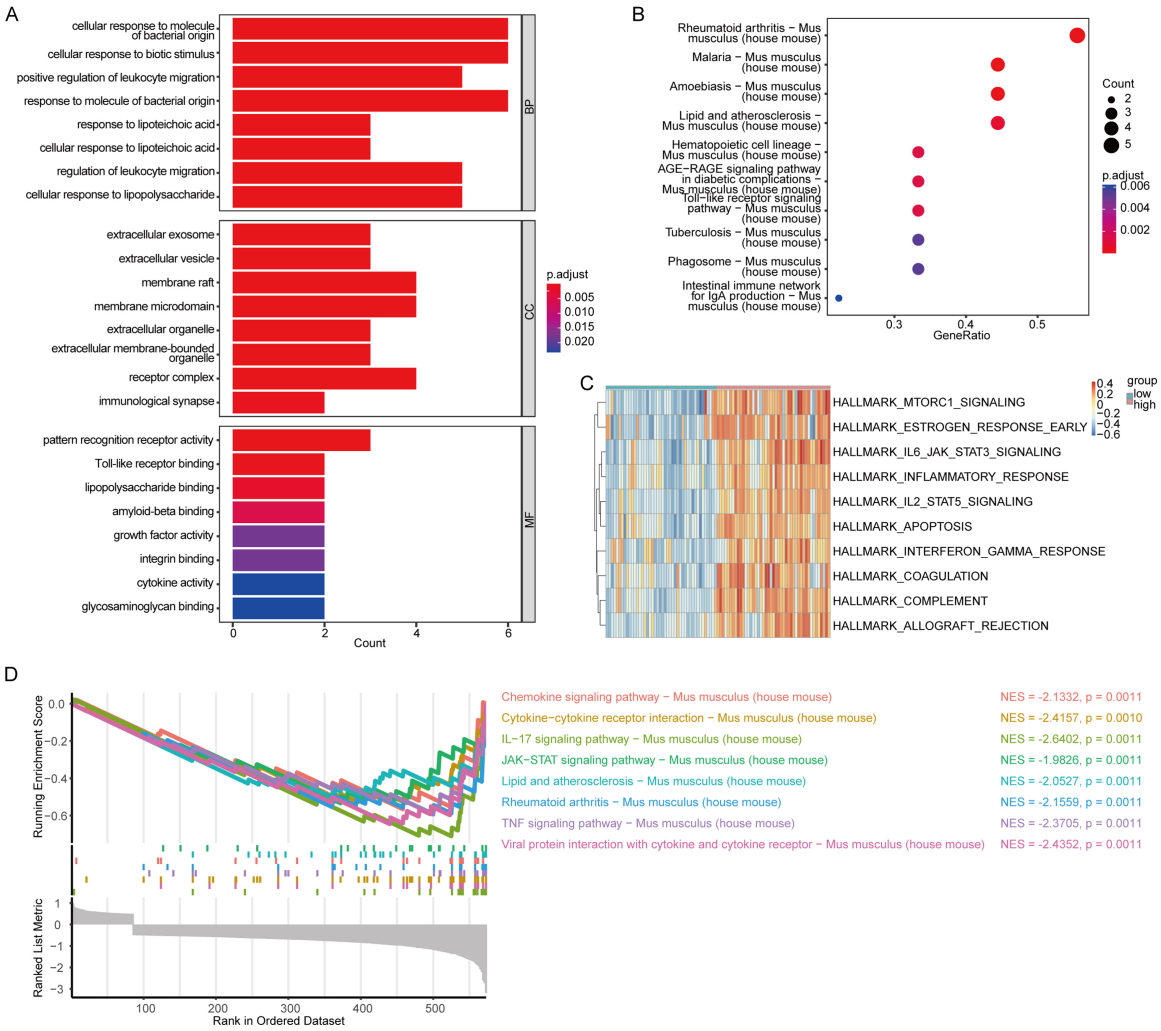
GO, KEGG, GSEA, and GSVA analysis of hub genes grouped based on ssGSEA score. **(A)** GO enrichment analysis of hub genes; **(B)** KEGG enrichment analysis of hub genes; **(C)** heatmap of GSVA; **(D)** specific enrichment of GSEA. Statistical significance was set at *p*-value <0.05.

Enrichment analyses GO and KEGG were performed on hub genes. GO analysis illustrated that these genes were primarily enriched in BP related to cellular responses to biotic stimuli, positive regulation of leukocyte migration and others. For CCs, they were primarily associated with extracellular exosomes, extracellular vesicles, and membrane rafts. Regarding MFs, they were enriched in pattern Toll-like receptor binding, lipopolysaccharide binding, and other MFs (Figure 7A). The complete GO enrichment analysis results were shown in Supplementary Table S8. Additionally, KEGG analysis uncovered that the hub genes were related to rheumatoid arthritis, malaria, amoebiasis, and others in *Mus musculus* (house mouse) (Figure 7B). The KEGG enrichment analysis results are presented in Supplementary Table S9.

To explore the mh. All. V2023.1. Mm. Symbols reference gene set pathways in the IS high-score and low-score groups, we calculated the hub gene scores for each IS sample using the ssGSEA algorithm. This allowed us to represent the level of ischemic cerebral apoplexy in the samples. The IS samples were categorized into high- and low-score groups according to the median score. Subsequently, GSEA and GSVA were used to compare the two groups within the IS dataset. The GSVA results revealed that several pathways were more enriched in the high-scoring group than in the low-scoring group, including IL2 STAT5 signaling (*p* = 3.09E-20), complement activation (*p* = 1.37E-19), IL6 JAK STAT3 signaling (*p* = 1.29E-18), apoptosis (*p* = 6.36E-16), MTORC1 signaling (*p* = 3.99E-15), inflammatory response (*p* = 2.03E-14), rejection (*p* = 3.17E-14), coagulation (*p* = 1.58E-12), early estrogen response (*p* = 4.35E-11), and interferon-gamma response (*p* = 4.38E-11). Figure 7C shows the enrichment of the relevant pathways. The results are presented in Supplementary Table S10.

The GSEA results highlight the top eight results with the smallest *p*-values, which include the following for *Mus musculus* (house mouse): lipid and atherosclerosis, JAK–STAT signaling pathway and others. Figure 7D illustrates the specific enrichment of the relevant pathways. The full GSEA enrichment analysis results are presented in Supplementary Table S11.

### 3.7 Immune infiltration analysis based on CIBERSORT

**Figure 8:**
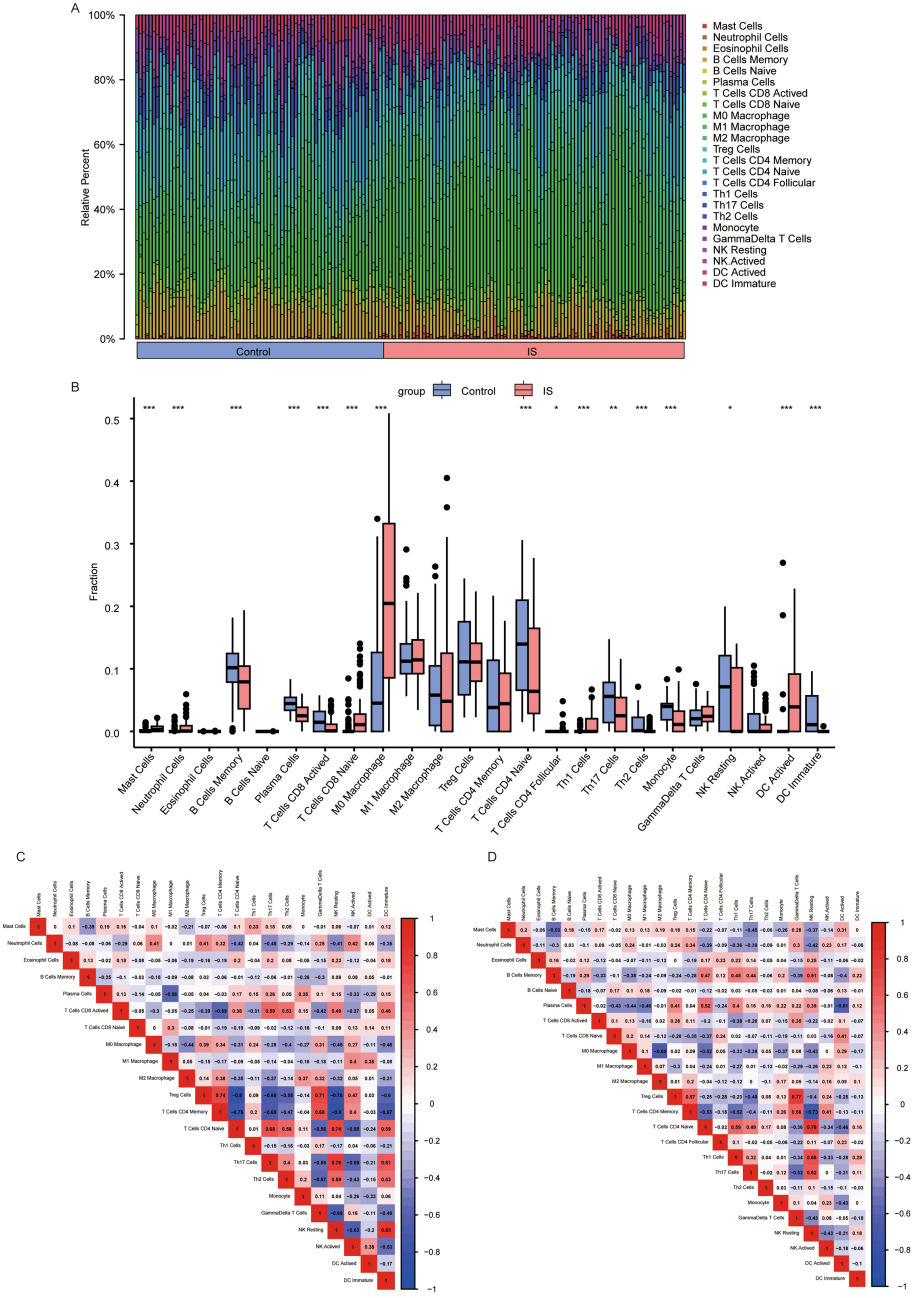
Analysis of 24 cellular immune infiltrates based on CIBERSORT. **(A)** Panorama of infiltration of 24 immune cells; **(B)** differences in each immune cell type and immune function (*: *p* < 0.05; * *: *p* < 0.01; ***: *p* < 0.001). Heatmap of correlations between immune cells based on CIBERSORT. **(C)** heatmap of relationship between immune cells in the control group; **(D)** heatmap of relationship between immune cells in the IS group. An absolute correlation coefficient r above 0.8 indicates a strong correlation, while values between 0.3 and 0.8 suggest a weak correlation. Coefficients below 0.3 imply no correlation. IS, ischemic stroke.

First, we employed CIBERSORT to analyze the infiltration of 25 types of immune cell infiltrations in the IS dataset. After excluding columns with zero values, we identified the following immune cell types: mast cells, neutrophils, eosinophils, memory B cells, naive B cells, plasma cells, active CD8 T cells, naive CD8 T cells, M0 Macrophage, M1 Macrophage, M2 Macrophage, regulatory T (Treg) cells, memory CD4 T cells, naive CD4 T cells, follicular CD4 T cells, Th1 cells, Th17 cells, Th2 cells, monocytes, gamma delta T cells, resting NK cells, active NK cells, activated dendritic cells, and immature dendritic cells. The infiltration levels of these 24 immune cell types were compared between the control and IS groups (Figures 8A,B). We found that the infiltration of naïve CD4 + T cells in the control group was higher than that in the IS group (*p* < 0.001). We analyzed and visualized the correlation between the 24 types of immune cells in the control and IS groups (Figures 8C,D). In the control group, the highest affirmative association was observed between immature dendritic cells and resting NK cells (*r* = 0.83, *p* = 9.66E-07). The inverse association between Treg cells and naive CD4+ T cells (*r* = −0.8, *p* = 7.10E-18), as well as the adverse relationship between CD4 + memory T cells and resting NK cells (*r* = −0.8, *p* = 9.78E-18), was stronger in the control group than in the IS group. The positive relation between naive CD4+ T cells and resting NK cells was strongest in the IS group (*r* = 0.78, *p* = 4.49E-19). Similarly, the inverse relationship between CD4+ memory T cells and resting NK cells was strongest in the IS group (*r* = −0.73, *p* = 7.10E-18) (*p* = 7.66E-16).

### 3.8 Identification of molecular subtypes of IS based on feature genes

**Figure 9:**
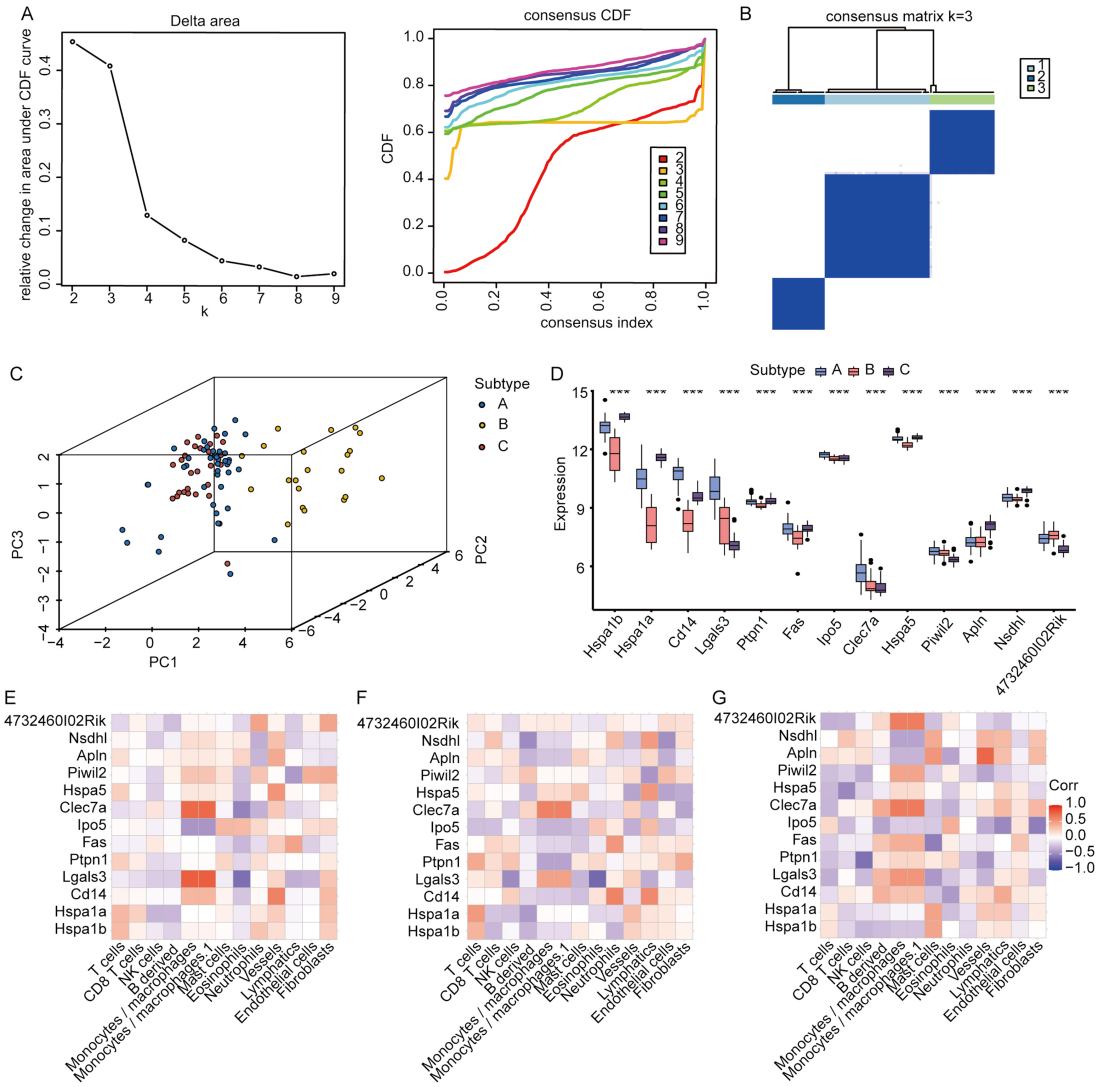
Identification of molecular subtypes of IS disease according to the IS dataset. **(A)** Selection of k value in the clustering process; **(B)** clustering heatmap when k = 3; **(C)** PCA analysis results of various IS molecular subtypes; **(D)** expression distribution of core genes among different IS molecular subtypes; **(E)** relationship between immune cells and feature genes of IS subtype A; **(F)** relationship between immune cells and feature genes in IS subtype B; **(G)** Relationship between immune cells and feature genes of IS subtype C (*: *p* < 0.05; **: *p* < 0.01; ***: *p* < 0.001. An absolute correlation coefficient r above 0.8 indicates a strong correlation, while values between 0.3 and 0.8 suggest a weak correlation. Coefficients below 0.3 imply no correlation).

We conducted a consistent clustering analysis on the IS dataset samples utilizing the 13 feature genes described above. Figure 9A shows the process of cluster k-value selection for the IS dataset. Subsequently, a k-value of 3 was selected as the clustering result, dividing the IS dataset samples into three disease subtypes: A, B, and C. The clustering results are presented as a heat map (Figure 9B). Subsequently, through PCA, we showed the distribution of the different subtypes (Figure 9C). The PCA results indicated significant differences among the three IS molecular subtypes. Next, we compared the variations in the expression of feature genes among the various IS molecular subtypes. We subsequently found that HSPA1B expression was the highest in subtype C, HSPA1A was the highest in subtype C, CD14 was the highest in subtype A, LGALS3 was the highest in subtype A, and PTPN1 was the highest in subtype C. The highest expression was observed for FAS in subtype A, IPO5 in subtype A, CLEC7A in subtype A, HSPA5 in subtype C, PIWIL2 in subtype A, APLN in subtype C, and NSDHL in subtype C. The expression of 4732460102RIK was the highest in subtype B (Figure 9D). We subsequently conducted an MCP-based immune infiltration analysis of the IS dataset to estimate the absolute abundance of each sample in the following cells: T cells, CD8+ T cells, NK cells, B-derived cells, monocytes/macrophages, monocytes/macrophages.1, mast cells, eosinophils, neutrophils, vessels, lymphatics, endothelial cells, and fibroblasts. The relationship between the expression of feature genes and immune abundance of MCP in disease subtypes A, B, and C was calculated. In subtype A (Figure 9E), LGALS3 exhibited the strongest positive correlation with monocytes/macrophages and monocyte/macrophage 1 cells (*r* = 0.79, *p* = 4.84E-10), as well as a strong positive connection to eosinophils (*r* = −0.62, *p* = 4.84E-10). Similarly, CLEC7A showed a strong positive relation to monocytes/macrophages (*r* = 0.74, *p* = 2.76E-08) and a negative relationship with eosinophils (*r* = −0.52, *p* = 4.66E-04). In subtype B (Figure 9F), CLEC7A was positively correlated with monocytes/macrophages and monocyte/macrophage.1 cells (*r* = 0.62, *p* = 2.60E-03), while CD14 exhibited a positive association with neutrophils (*r* = 0.61, *p* = 4.66E-04) and lymphatics (*r* = 0.58, *p* = 5.40E-03). Additionally, LGALS3 demonstrated the highest negative connection to eosinophil count (*r* = −0.65, *p* = 1.53E-03). In subtype C (Figure 9G), APLN exhibited the strongest affirmative relation to vessels (*r* = 0.75, *p* = 1.10E-05) and CLEC7A exhibited the strongest positive association with monocytes/macrophages. Additionally, 4732460102RIK was positively related to monocytes/macrophages and monocytes/macrophages.1 (*r* = 0.64, *p* = 3.8E-04) cells. PTPN1 exhibited the highest adverse association with NK cells (*r* = −0.61, *p* = 9.8E-04), FAS exhibited the tightest negative linkage to mast cells (*r* = −0.55, *p* = 3.8E-03), and IPO5 exhibited the strongest negative relation to fibroblasts (*r* = −0.55, *p* = 3.3E-03).

### 3.9 Correlation analysis between hub genes and immune cells in different molecular subtypes

**Figure 10:**
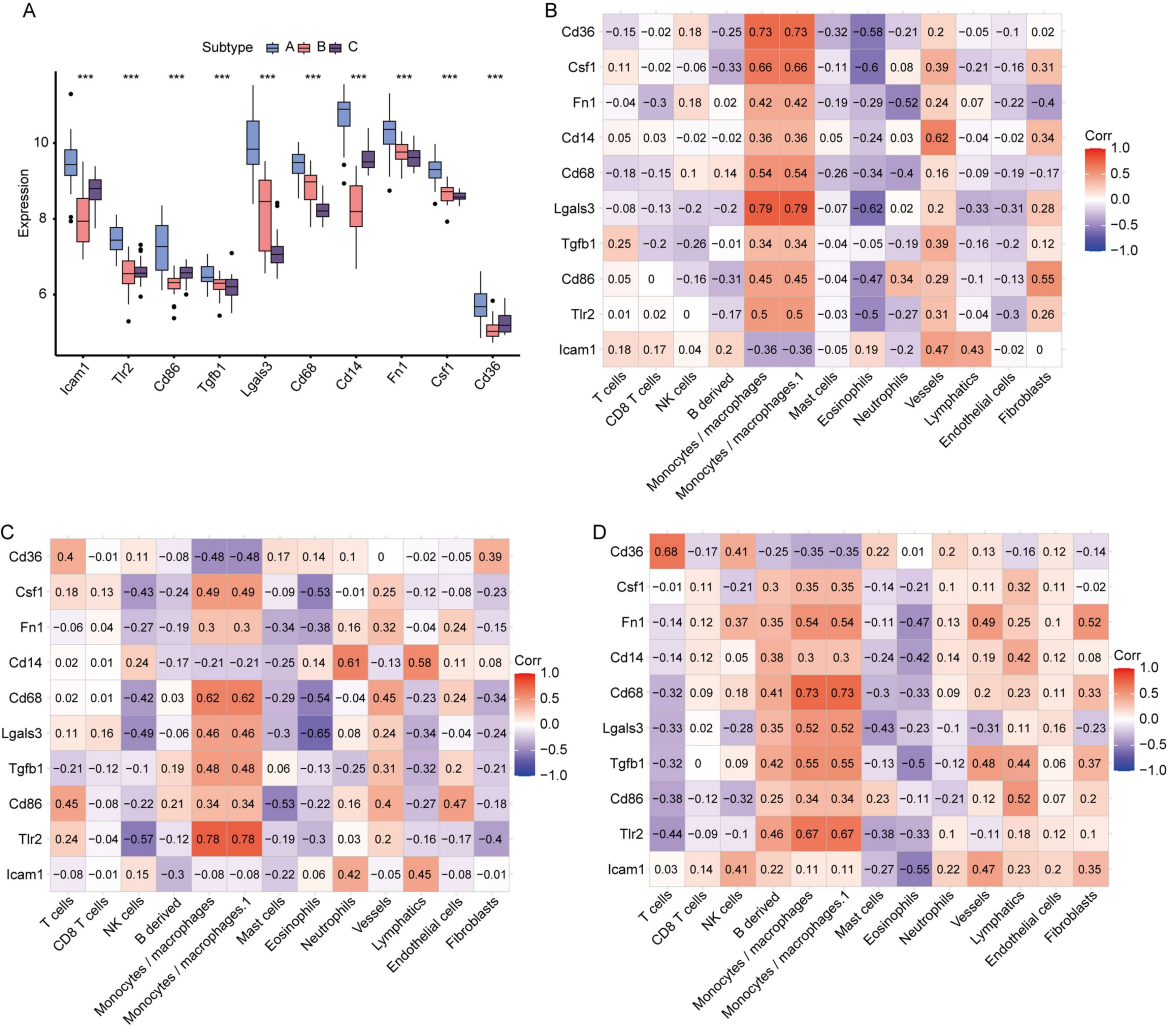
Correlation between hub genes and MCPcounter among different IS subtypes. **(A)** Expression of hub genes among different IS molecular subtypes; **(B)** relationship between immune cells and hub genes in IS subtype A; **(C)** correlation between immune cells and hub genes in IS subtype B; **(D)** relationship between immune cells and hub genes in IS subtype C (*: *p* < 0.05; **: *p* < 0.01; ***: *p* < 0.001. An absolute correlation coefficient r above 0.8 indicates a strong correlation, while values between 0.3 and 0.8 suggest a weak correlation. Coefficients below 0.3 imply no correlation).

Comparing the expression of hub genes among the aforementioned disease molecular subtypes (Figure 10A), we found that all hub genes—namely ICAM1, FN1, CD36, TLR2, CD86, LGALS3, CSF1, CD14, CD68, and TGFB1—exhibited the highest expression levels in subtype A. Using the immune cell infiltration obtained via MCP, we assessed the association between hub gene expression and immune cell abundance in disease subtypes A, B, and C. In subtype A (Figure 10B), LGALS3 exhibited the strongest positive correlation with monocytes/macrophages and monocytes/macrophages.1 (*r* = 0.79, *p* = 4.84E-10) cells. CD36 was positively correlated with monocytes/macrophages and monocytes/macrophages.1 (*r* = 0.73, *p* = 4.84E-10). CD36 and CSF1 had a strong affirmative relationship with monocytes/macrophages and monocytes/macrophages.1 (*r* = 0.66, *p* = 1.70E-06), while LGALS3 exhibited a strong positive relation to eosinophils (*r* = −0.62, *p* = 0.73). The negative correlations between CSF1 and eosinophils (*r* = −0.6, *p* = 2.69E-05) and between CD36 and eosinophils (*r* = −0.58, *p* = 5.86E-05) were notably strong. In subtype B (Figure 10C), TLR2 showed the strongest correlation with monocytes/macrophages and monocytes/macrophages.1 (*r* = 0.78, *p* = 3.31E-05) cells. The positive correlation between CD68 and monocytes/macrophages and monocytes/macrophages.1 (*r* = 0.62), as well as between CD14 and neutrophils (*r* = 0.61, *p* = 2.9E-03) was significant. LGALS3 exhibited the highest negative relation to eosinophils (*r* = −0.65, *p* = 1.5E-03). In subtype C (Figure 10D), CD68 was associated with monocytes/macrophages and monocytes/macrophages.1 (*r* = 0.73, *p* = 2.73E-05), CD36 was related to T cells (*r* = 0.68, *p* = 2.73E-05), CD36 was connected to eosinophils (*r* = 0.68, *p* = 1.5E-03) (*p* = 1.24E-04), and TLR2 positively correlated with monocytes/macrophages and monocytes/macrophages.1 (*r* = 0.67, *p* = 1.6E-04) cells. The tightest inverse relation was identified between ICAM1 and eosinophils (*r* = −0.55, *p* = −3.75E-03).

### 3.10 Heterogeneity of single cell data

**Figure 11:**
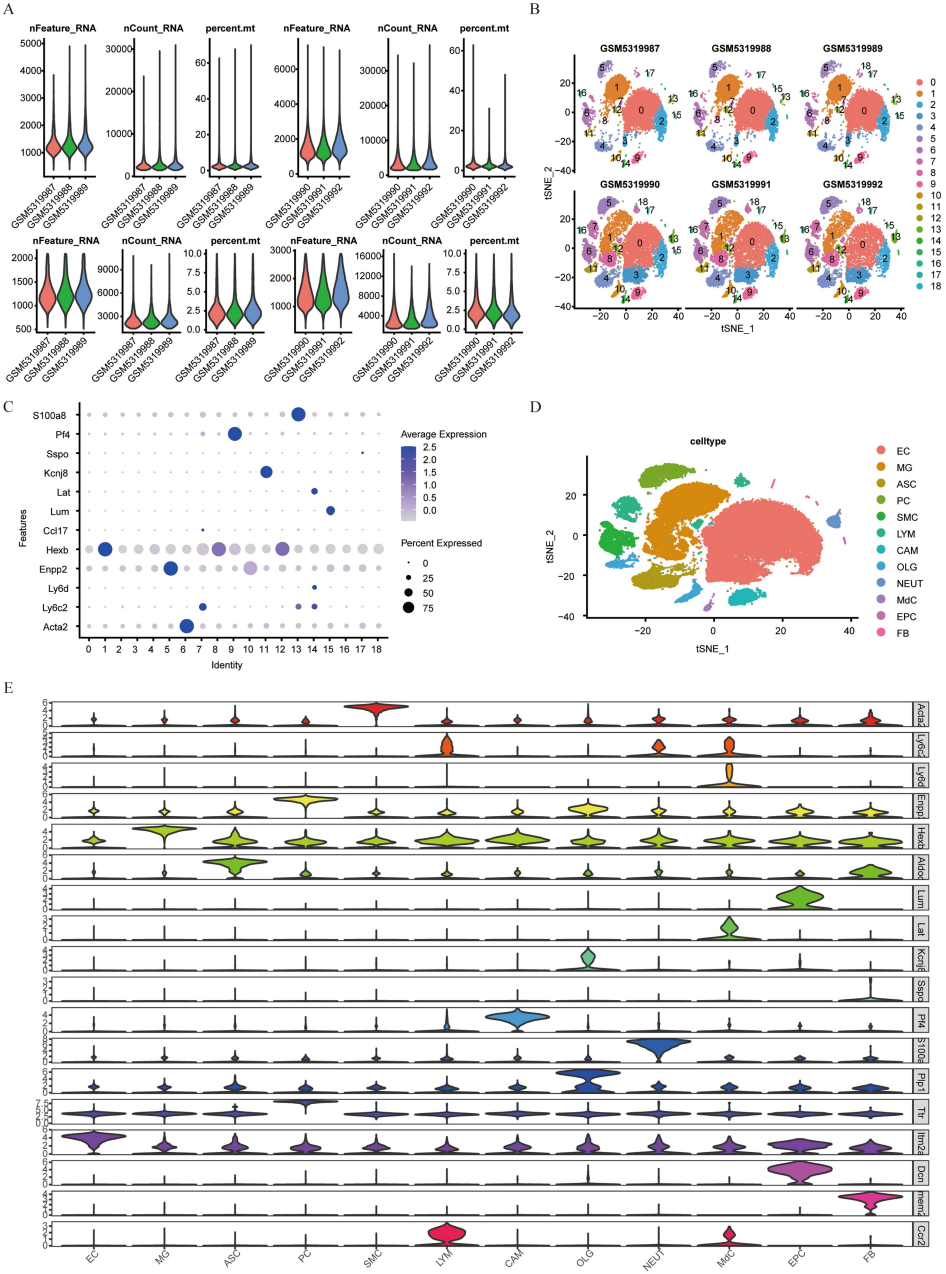
Quality control and clustering annotation of single-cell data. **(A)** Gene count, RNA count, and percentage of mitochondria in samples before quality control; **(B)** gene count, RNA count, and percentage of mitochondria in samples after quality control; **(C)** t-SNE cluster map of 19 cell populations in different samples; **(D)** t-SNE cluster map of 12 cell types; **(E)** Violin plot of all marker genes expressed between different cells.

Quality control was performed on single-cell IS samples (GSM5319990, GSM5319991, and GSM5319992, referred to as IS1, IS2, and IS3) and control samples (GSM5319987, GSM5319988, and GSM5319989; labeled CL1, CL2, and CL3) using the Seurat package. Cells with mitochondrial gene content over 15%, fewer than 200 features, or Unique Molecular Identifiers (UMI) greater than 20,000 were excluded (Figure 11A). The remaining cells were then grouped into 19 single-cell subsets using t-SNE (t-Distributed Stochastic Neighbor Embedding) clustering (Figure 11B). Marker genes from the literature were used for annotation (Figure 11C). After removing two cell subsets, 16 and 18, which were not significantly expressed, cluster 6 comprised vascular SMC, and cluster 17 comprised perivascular FB. Cluster 9 included CAM, Cluster 14 included MdCs, and Clusters 0, 2, and 3 included EC. Cluster 15 comprised EPC, and clusters 1, 8, and 12 included MG. Cluster 13 comprised NEUT, cluster 4 comprised ASC, and clusters 10 and 11 comprised OLG. Cluster 7 comprised LYM, and cluster 5 comprised PC. This annotation resulted in a clustering map of 12 cell types for each sample (Figure 11D).

We then identified the expression of marker genes in 19 single-cell subsets of IS single-cell samples from different cell clusters. A bubble plot illustrated the expression of marker genes in various cell clusters (Figure 11C). At the same time, using a violin plot, we observed large differences in marker gene expression between different cell clusters. In Figure 11E, ACTA2 gene was markedly expressed in cluster 6, LY6C2 in cluster 7, and LY6D in cluster 14. ENPP2 was abundantly expressed in clusters 5 and 10, while HEXB was strongly expressed in clusters 1, 8, and 12. LUM was prominently expressed in cluster 15, LAT in cluster 14, and KCNJ8 in cluster 11. Additionally, SSPO was markedly expressed in cluster 17, PF4 in cluster 9, and S100A8 in cluster 13.

### 3.11 Analysis of the difference and proportion of single-cell groups

**Figure 12:**
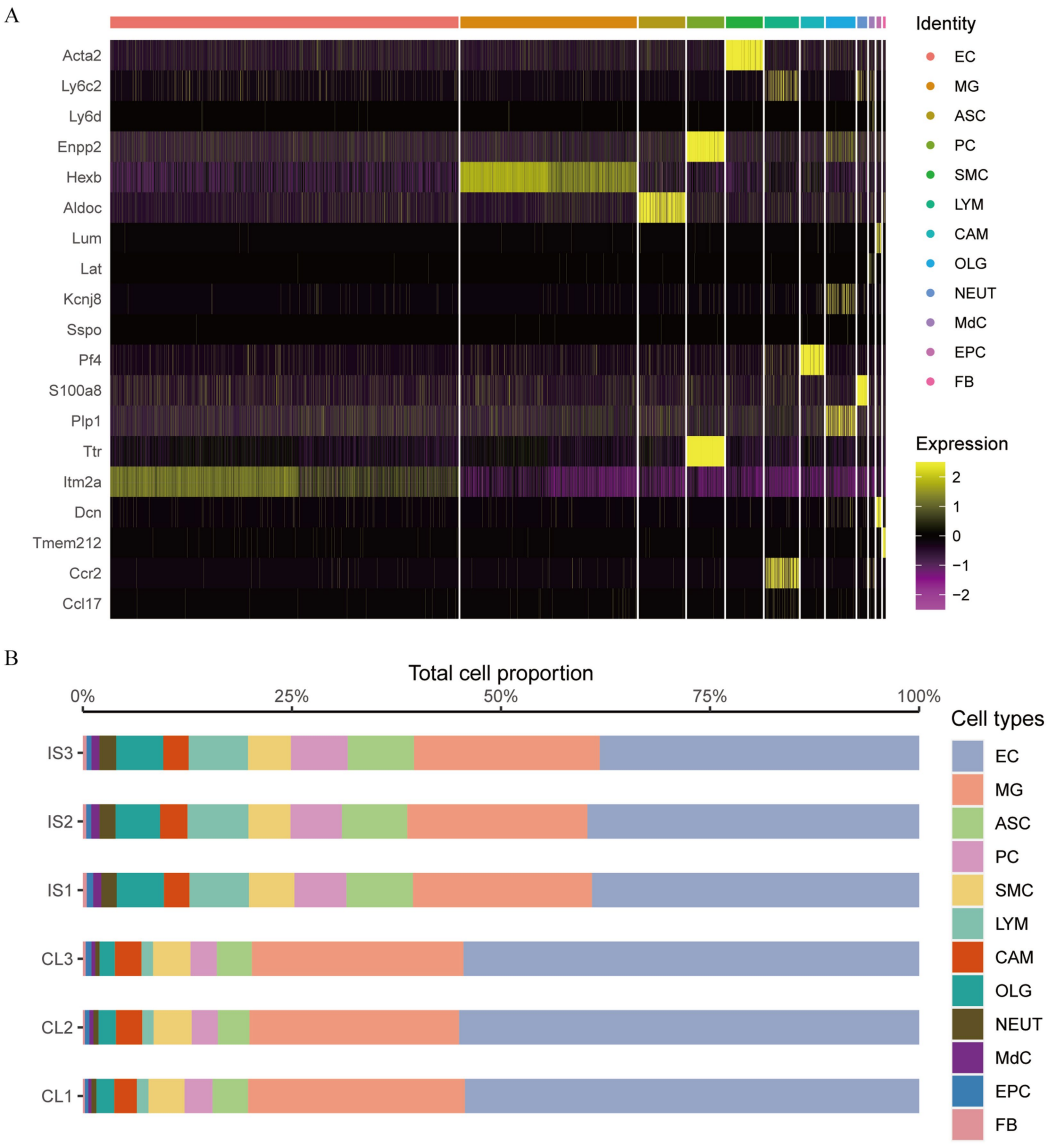
The proportions of EC and MG cells. **(A)** Heat map of marker genes between cell clusters in cerebral ischemia samples; **(B)** proportion of cell groups in the cerebral ischemia group and the control group.

We analyzed the expression variations among cell populations within the IS samples and visualized the top 20 diagnostic markers using a heatmap (Figure 12A). ITM2A and ARG1 were abundantly expressed in EC cells. HEXB gene was markedly expressed in MG cells. ALDOC gene was strongly expressed in ASC cells. ENPP2 and TTR genes were markedly expressed in PC cells. ACTA2 gene was strongly expressed in SMC cells. LY6C2 and CCR2 genes were markedly expressed in LYM cells. PF4 was strongly expressed in CAM cells. KCNJ8, PLP1, and DCN genes were markedly expressed in OLG cells. S100A8 gene was markedly expressed in NEUT cells. LY6C2 was markedly expressed in MdC. LUM and DCN were prominently expressed in EPC cells. TMEM212 gene was highly expressed in FB cells.

We also estimated the disparity in the proportion of cell groups in cerebral ischemia and normal samples and found that the proportion of cells in the two groups was basically the same: EC cells had the highest proportion, followed by MG cells, while FB cells had the lowest proportion (Figure 12B). The proportions of EC and MG cells were greater in the control group than those in the IS group.

### 3.12 Score of feature genes in single cell data and subgrouping of cell subtypes

**Figure 13:**
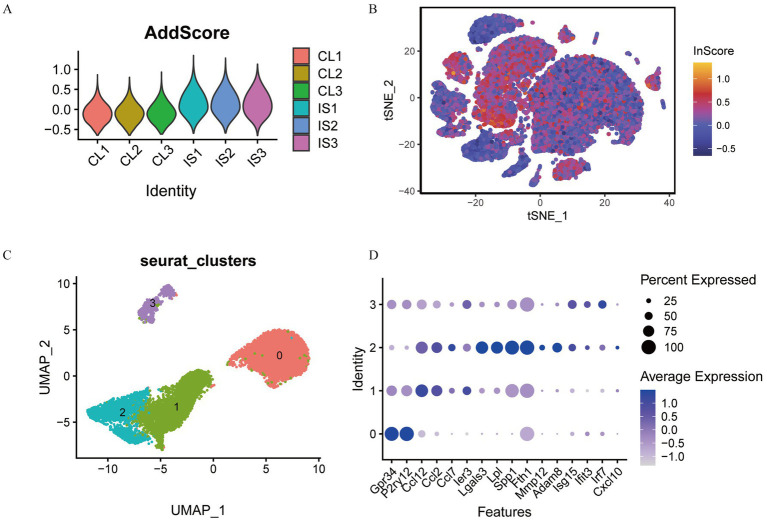
Cell subtype grouping based on feature gene score. **(A)** Feature gene score of each sample; **(B)** visualization of feature gene scores in t-SNE cell groups; **(C)** t-SNE cluster map of MG cell subtypes; **(D)** bubble plot of MG cell marker genes.

To test the expression score of transcriptomic feature genes within single-cell datasets, we utilized the AddModuleScore function from the Seurat package. This allowed us to evaluate the expression of HSPA1B, HSPA1A, CD14, LGALS3, PTPN1, FAS, IPO5, CLEC7A, HSPA5, PIWIL2, APLN, NSDHL, and 4732460I02RIK in each cell population. As a result, we obtained scores reflecting the expressions of these feature genes. The scores of each sample were presented in Figure 13A, demonstrating that the IS group has a greatly increased score than that of the CL group. We visualized the score of each cell in the t-SNE cluster map, as illustrated in Figure 13B, clearly indicating that MG cells have the highest score combined with the corresponding t-SNE cluster map Figure 11D.

Utilizing the uniform manifold approximation and projection technique, we identified and annotated four distinct subtypes of microglia based on previously described marker genes. Ultimately, we classified the microglia into four subsets: MG0, MG1, MG2, and MG3 (Figure 13C). The expression of marker genes in the cell subsets was presented with a bubble plot (Figure 13D). We found that MG0 cells mainly expressed GPR34 and P2RY12 genes. MG1 cells primarily expressed CCL12, CCL2, CCL7, and IER3 genes. MG2 cells mainly expressed LGALS3, LPL, SPP1, FTH1, and MMP12 genes. MG3 cells mainly expressed ISG15 and IRF7 genes.

### 3.13 MG cell population for pseudo-timing analysis and cell communication analysis

**Figure 14:**
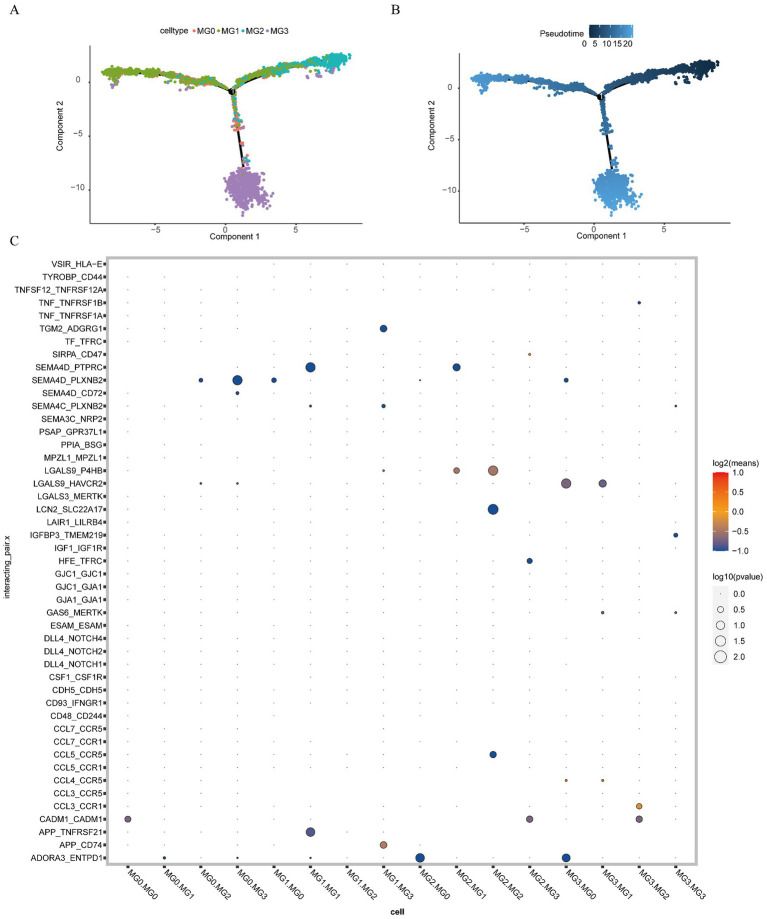
Pseudo-timing analysis and cell communication analysis. **(A)** Developmental trajectories of MG cell subtypes (different colors represent different cell subtypes); **(B)** MG cell subtype differentiation and development time sequence diagram (colors range from dark to light to indicate the progression over time); **(C)** ligand interaction bubble diagram between MG cell subsets (the color of the bubbles transitions from blue to red, indicating the strength of interaction, with blue representing weak interactions and red representing strong interactions. Additionally, the size of the bubbles varies from large to small, reflecting the significance of the interactions, with larger bubbles indicating stronger significance).

We explored the developmental trajectories of the four types of MG cells using quasi-temporal analysis and demonstrated the developmental trajectories of MG cells through differentiation and development trajectory plots (Figure 14A) and differentiation and development sequence plots (Figure 14B). Developmental trajectories of the subtypes were inferred as MG2, MG1, MG0, and MG3.

To explore the receptor-ligand interactions among MG cell subsets, we presented the strength of the receptor-ligand interactions among different cell subsets in the form of bubble plots (Figure 14C). We found that APP_CD74 receptor-ligand pairs exhibited strong interaction strength between MG1 and MG3 cells. Additionally, LGALS9_P4HB receptor-ligand pairs demonstrated high interaction strength between MG2 and MG1 cells. The interaction between LGALS9_P4HB receptor-ligand pairs was stronger in MG2 cells than in MG2 cells. Furthermore, CCL3_CCR1 receptor-ligand pairs exhibited a strong interaction strength between MG2 and MG3 cells. The detailed results are presented in Supplementary Table S12.

### 3.14 Enrichment analysis of single cell data

**Figure 15:**
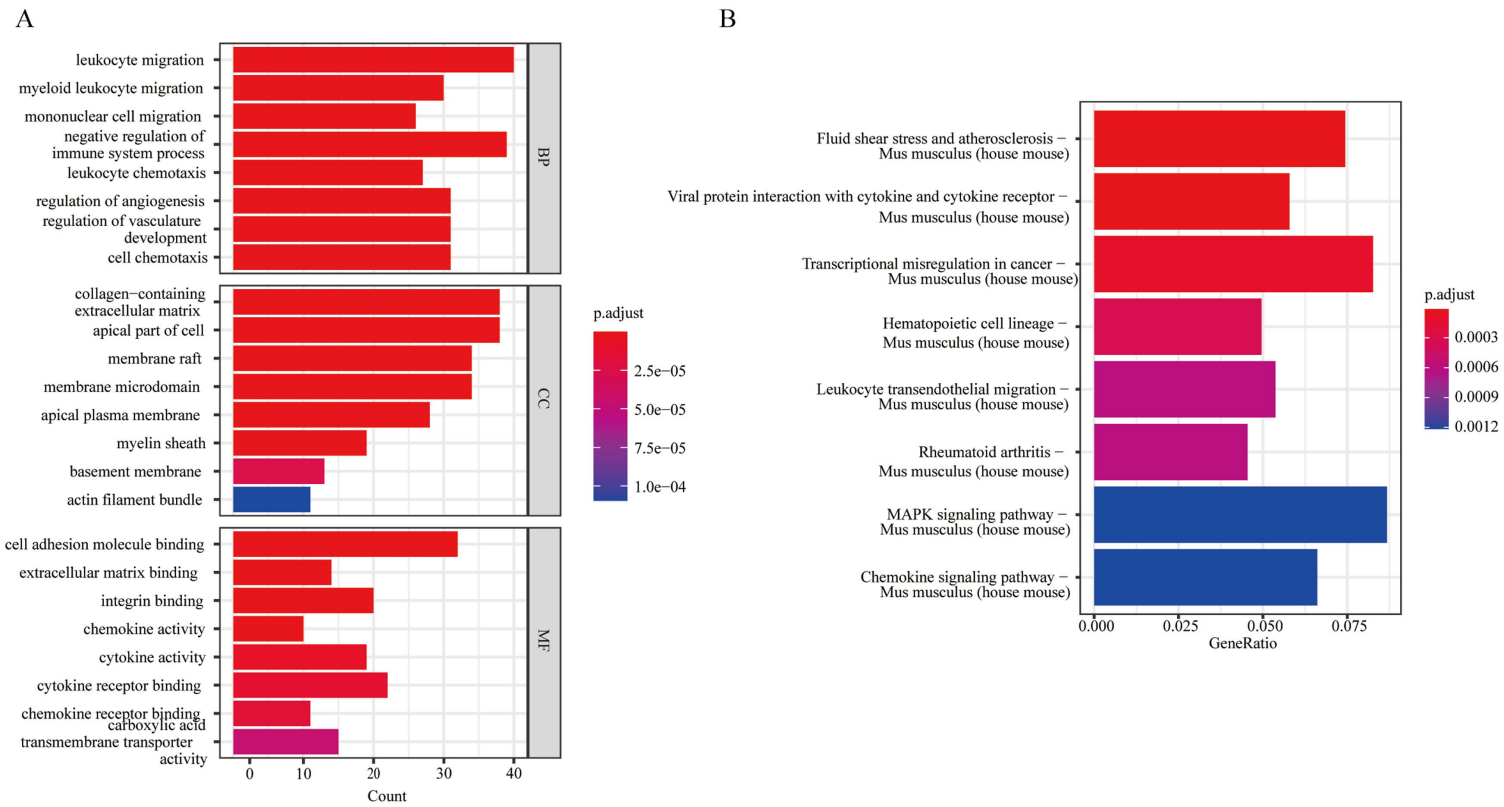
GO and KEGG analysis of differentially expressed genes between cells. **(A)** GO enrichment analysis of MG cells; **(B)** KEGG enrichment analysis of MG cells (statistical significance was set at *p* < 0.05). MG, microglia.

Differential gene analysis was conducted among different subgroups of MG cells, resulting in the identification of 483 DEGs selected based on the criteria of |log2FoldChange| > 0.5 and *p*-value <0.05. We performed GO and KEGG enrichment analyses of the DEGs. GO analysis indicated that the 483 DEGs were primarily enriched in BP, such as myeloid leukocyte migration and others. Additionally, they were significantly enriched in CCs, including apical part of the cell, membrane rafts and others. For MF, they were primarily associated with extracellular matrix binding, integrin binding and others (Figure 15A). Complete GO enrichment analysis is listed in Supplementary Table S13. KEGG analysis revealed that hub genes correlated to several pathways, including fluid shear stress and atherosclerosis in *Mus musculus* (house mouse) and others (Figure 15B). The KEGG enrichment analysis results are presented in Supplementary Table S14.

## 4 Discussion

IS is the primary cause of morbidity and mortality worldwide (2). The complex and poorly understood pathophysiology of IS has limited advancements in prognosis and prevention. Exosomes facilitate intercellular communication by shuttling genetic information and proteins between cells, playing a crucial role in various cellular processes. Moreover, following brain injury, exosomes are generated by brain cells and activate various responses. Thus, specific exosomes or their cargos can be utilized for the clinical assessment of IS (47, 48). Following IS, neuroinflammation triggers the release of pro-inflammatory factors after the activation of various cells, including glial cells, leukocytes, and monocytes (49). Following cerebral ischemia, microglia display changes in morphology and phenotype (50). M1 microglia secrete pro-inflammatory factors, worsening inflammation, whereas M2 microglia exert anti-inflammatory effects during hypoxic and glucose-deprived ischemic injury (51). Different microglial subtypes have distinct functions that vary over time and under different conditions.

From a clinical relevance perspective, our analysis suggests that these exosome-related genes may be associated with the disease progression, inflammatory response, or neuronal injury repair in ischemic stroke. Therefore, these genes could potentially serve as biomarkers for early diagnosis or risk assessment of the disease (47, 48). As carriers of bioactive molecules, exosomes and their associated genes may have significant application value in developing new therapeutic strategies. For instance, by regulating the expression of these genes, it may be possible to influence the biological functions of exosomes, thereby improving the pathological processes of ischemic stroke. Additionally, using engineered exosomes to deliver specific RNA or proteins could become a future therapeutic strategy. Furthermore, intervention strategies targeting these genes, such as RNA interference or monoclonal antibody therapy, may offer new treatment options for patients with ischemic stroke.

Recent studies have highlighted the complex interactions between exosomes and neuroinflammation (52). On one hand, exosomal miRNAs negatively regulate target gene; for example, M2 microglia-derived exosomes alleviate ischemic injury and increase neuronal viability by downregulating their targets (53). On the other hand, exosomal miRNAs act as ligands that bind to receptors; for instance, exosomal miR-21 and miR-29a bind to TLRs, inducing NF-κB activation and the release of pro-inflammatory cytokines (54).

Hence, we focused on integrating multi-omics data and advanced analytical methods to explore the mechanisms of IS and its association with exosome-related genes. In this study, we identified 13 feature genes and 10 hub genes and conducted enrichment analysis to identify their biological mechanisms. The infiltration status of the 24 immune cells was determined by immune infiltration analysis, and we examined their correlations with feature genes and hub genes across different molecular subtypes of IS. Single-cell sequencing revealed heterogeneity among MG subpopulations with significant differences in marker expression. The developmental trajectories of these subtypes were inferred as MG2, MG1, MG0, and MG4 cells.

LGALS3, also known as GALECTIN-3, participates in various cellular processes (55). Differences in the effects of LGALS3 on IS are likely attributable to the time-dependent and context-specific nature of its actions (56). In this research, LGALS3 was identified as a hub gene, and qPCR revealed that its expression was significantly elevated. This corroborates earlier research results that investigated the expression of core genes related to neuroinflammation in the IS (57). Moreover, our study identified that LGALS3 interacts with various proteins, including CD36, to form networks. Similarly, a study on demyelination observed that LGALS3 can modulate microglial cells via the PPARγ-CD36 pathway (58). Additionally, recent research has investigated the therapeutic potential of LGALS3 by conjugating it with glucosamine to mitigate inflammatory responses following IS (59). Given the complexity and uncertainty of LGALS3 in IS, impactful findings have yet to be realized. However, as a biomarker for diagnosis and prognosis, LGALS3 demonstrates substantial advantages. CD14 and TLR2 were also identified as hub genes in our study, and their expression levels were confirmed to be significantly elevated by qPCR. As a coreceptor of TLR, CD14 has been shown to modulate immune responses via TLR2-derived peptides, which might be a prosperous strategy (60). Moreover, specific antibodies could facilitate inflammation via activating the TLR2/CD14 receptor complex, which contributes to atherosclerosis-related complications (61). Therefore, we hypothesize that the elevated expression of CD14 and TLR2 might be important biomarkers for the exacerbation of IS, warranting further extensive experimental validation.

IS leads to neuronal necrosis and triggers inflammatory responses. Cytokines serve as the primary mediators of this inflammatory reaction, with both pro-inflammatory and anti-inflammatory cytokines being released in the ischemic brain tissue, exerting dual effects on cell survival (62). The JAK/STAT pathway is an essential participant in the cellular response to cytokines (63). However, the function of JAK/STAT signaling remains controversial. LGALS3 can enhance the phosphorylation of JAK2, STAT3 and STAT5, thereby promoting glial cells to generate increased levels of pro-inflammatory mediators and exhibit activated characteristics (64). Additionally, the application of inhibitors to block the phosphorylation of JAK2 and its downstream molecules, STAT1 and STAT3, significantly reduces the expression of renal ICAM-1, thereby alleviating the damage associated with renal ischemia–reperfusion (65). In our study, the JAK/STAT signaling pathway was enriched in GSEA and GSVA. Notably, LGALS3 and ICAM-1, identified as hub genes, showed elevated expression in the IS group using qPCR. These findings suggest that modulation of the JAK/STAT pathway might be a promising candidate target for early intervention in patients with IS. Despite the current research indicating the protective effects of JAK/STAT in IS, such as the upregulation of miR-216a targeting JAK2 to mitigate ischemic neuronal injury (66), there remains a pressing need for further investigation.

Following acute IS, immune mediators rapidly trigger pro-inflammatory signals that activate resident cells and facilitate the influx of diverse inflammatory cells into the ischemic region, thereby influencing brain injury. Therefore, immune analysis of exosomal genes in IS is of great significance. In our immune infiltration analysis, the infiltration of naïve CD4+ T cells was higher in the control group than in the IS group. This high infiltration indicates that naïve CD4+ T cells proliferate and differentiate into several effector subsets, such as conventional T helper and Treg cells, after IS. However, specific regulatory mechanisms underlying this process remain unclear. Notably, Treg cells and naive CD4+ T cells were negatively correlated. Despite the controversy surrounding Treg dynamics in IS, their neuroprotective role remains unclear. A previous study has shown that Treg cells increased the infarction area following IS and worsened neurological function in mouse experiments (67). In contrast, other researchers have found that Treg cells act as neuroprotective modulators of brain inflammation following IS and can prevent the growth of secondary infarctions (68). Considering the immunosuppressive role of Treg cells, it may represent a potential strategy for targeted IS therapy in the future. Furthermore, through our identification of molecular subtypes in IS, we found that LGALS3 and CD14 are positively correlated with monocytes/macrophages and neutrophils, respectively, as previously reported. Previous studies on myocardial infarction have demonstrated that Lgals3 can promote the recruitment of macrophages to the infarcted myocardium and exerts a vital role in the repair phase (69). Moreover, LGALS3 combines with glycoconjugates on the cell surface, promoting macrophage adhesion and chemotaxis toward damaged tissues (70). Likewise, CD14-regulated responses can recruit neutrophils and macrophages to activate TNF signaling (71). However, in the context of IS, these effects have been scarcely reported. These findings indicate that CD14 and LGALS3 may serve as immunotherapeutic targets for neuroinflammatory responses associated with IS, which continue to be a significant focus of research.

Based on the results of immune infiltration and single-cell analysis, the exosome-related feature genes identified in this study (LGALS3, CD14, TLR2) regulate the neuroinflammatory process following ischemic stroke through multidimensional mechanisms. LGALS3 may play a role in promoting the release of inflammatory factors and modulating microglial cell polarization. LGALS3 activates microglia via the JAK/STAT pathway (64), promoting the release of inflammatory factors, while also regulating peripheral neurons and immune cells through exosomal miRNA transmission. CD14 and TLR2 may activate the NF-κB pathway by recognizing damage-associated molecular patterns (DAMPs), thereby promoting the production of pro-inflammatory cytokines. Furthermore, exosomal miRNAs may regulate immune responses by targeting these genes. The high expression of LGALS3 in the MG2 microglial cell subset, which predominates early after stroke, suggests its potential role in promoting a pro-inflammatory environment, whereas MG0, in its resting state, may exert a protective effect.

MG, the resident immune cells in the brain, constitutes approximately 20% of glial cells. In their resting state, microglia are characterized by small cell bodies with broad, branching processes. Microglial cells are constantly active, continuously surveying the brain to preserve tissue integrity (72). Following ischemic events, these cells become rapidly activated within a few minutes, undergoing changes in both shape and function. As a result, various microglial subpopulations play distinct roles; however, the precise mechanisms driving these changes remain unclear. Our single-cell sequencing analysis revealed that MG cells constituted a significant proportion of the cell population in the samples. Additionally, the HEXB gene was highly expressed in these cells. Previous studies identified the specific promoter activity of HEXB in the MG and have aimed to develop new genetic tools to further investigate microglia functions in the CNS, recognizing HEXB as a consistently expressed core microglial gene (73, 74). Subsequently, by combining 13 feature genes related to exosomes, we identified four distinct MG subtypes, annotated as MG0, MG1, MG2, and MG3. Each subtype highly expressed different genes, indicating possible divergent roles in IS. Notably, MG2 cells were distinguished by the expression of genes related to inflammation and phagocytosis, including LGALS3, FTH1, LPL, and SPP1, which are markers of activated MG. In contrast, MG0 cells predominantly expressed the P2ry12 gene, a marker associated with relatively resting microglia. Our findings indicate that MG2 (highly active microglia) precedes MG1 (intermediate activity state) in developmental trajectories, with resting MG0 positioned last. This finding confirms that microglial activation in the IS not a straightforward transition from a resting to an active state but rather exists in a dynamic equilibrium, allowing for rapid and variable responses and multiple instances of reversal. Notably, a previous study reported that microglial development progressed from a resting state to a highly active state, using LASSO, SVM-RFE, and Boruta analyses combined with single-cell sequencing analysis (75), which contrasts with our findings. However, owing to the differences in research methods and processing conditions, these paradoxical results further suggest that microglial behavior may be relatively complex and diverse across different contexts and time points. Considering the complex biological activities and interaction mechanisms of MG, our study may also reveal a novel mechanism that requires further investigation.

## 5 Limitation

Although a detailed bioinformatics analysis was performed in our research, several potential constraints need to be recognized. First, our study relied primarily on publicly available datasets, which might introduce batch effects despite our efforts to mitigate them through normalization and batch-effect removal techniques. Moreover, our study lacks in-depth experimental validation, and the identified key genes and pathways still need to be confirmed through laboratory experiments to establish their biological relevance and potential as therapeutic targets. Finally, the absence of clinical validation indicates that the translational potential of our findings remains speculative. Future studies should address these limitations by incorporating larger, independent cohorts and conducting rigorous experimental and clinical validation. Future studies will require more comprehensive experiments to further validate the role of the identified genes in ischemic stroke (IS). This includes constructing overexpression and knockout models to assess their impact on neuronal/astrocyte survival, inflammation, and apoptosis in IS progression. Using the MCAO mouse model, key gene expression can be manipulated via AAV infection or shRNA interference, followed by evaluation of neuronal survival, inflammation, and behavioral changes. RNA-seq or single-cell sequencing can be employed to investigate the signaling pathways, while co-immunoprecipitation and ChIP-qPCR will explore interactions with miRNAs, transcription factors, and downstream signaling proteins.

In future studies, we recommend employing a multidimensional research strategy to further enhance the robustness of the conclusions and their clinical translational value. First, it is necessary to validate the expression stability and prognostic predictive efficacy of key genes (e.g., LGALS3, CD14) in independent external cohorts, while systematically analyzing their molecular functions in cell/animal models using lab techniques such as RT-qPCR and Western blot. Second, techniques such as immunohistochemistry and single-cell spatial transcriptomics should be employed to validate gene expression profiles at the protein level and across cell subpopulations, revealing their spatial dynamics in pathological processes such as blood–brain barrier disruption and neuronal pyroptosis. At the clinical translational level, integrating proteomics, metabolomics, and imaging data to establish multimodal predictive models is essential, along with prospectively tracking gene expression and neurological function scores (e.g., NIHSS). Finally, incorporating artificial intelligence algorithms to construct a dynamic predictive framework of “gene expression-immune microenvironment-clinical outcomes” will provide a cross-scale evidence chain for precise subtype classification and targeted therapy in ischemic stroke.

## 6 Conclusion

Conclusively, we offer a detailed analysis of the role of exosome-related genes in IS. Our findings identified exosome-related genes, elucidated their potential regulatory networks, and highlighted the roles of LGALS3 and CD14 in the development of IS.

## Data Availability

The original contributions presented in the study are included in the article/Supplementary material, further inquiries can be directed to the corresponding authors.

## Footnotes

^1^ https://www.ncbi.nlm.nih.gov/geo/

^2^ http://www.excarta.org/

^3^ https://string-db.org

^4^ https://starbase.sysu.edu.cn/

^5^ https://rna.sysu.edu.cn/chipbase/

^6^ https://cibersort.stanford.edu/

^7^ https://www.exocarta.org/
